# Risk factors for falls among older adults in India: A systematic review and meta‐analysis

**DOI:** 10.1002/hsr2.637

**Published:** 2022-06-21

**Authors:** Isha Biswas, Busola Adebusoye, Kaushik Chattopadhyay

**Affiliations:** ^1^ Division of Epidemiology and Public Health, School of Medicine University of Nottingham Nottingham United Kingdom; ^2^ The Nottingham Centre for Evidence‐Based Healthcare: A JBI Centre of Excellence Nottingham United Kingdom

**Keywords:** falls, India, meta‐analysis, older adults, risk factors, systematic review

## Abstract

**Background and Aim:**

Falls are common among older adults in India. Several primary studies on its risk factors have been conducted in India. However, no systematic review has been conducted on this topic. Thus, the objective of this systematic review was to synthesize the existing evidence on the risk factors for falls among older adults in India.

**Methods:**

JBI and Preferred Reporting Items for Systematic Reviews and Meta‐Analyse guidelines were followed, and two independent reviewers were involved in the process. This review included observational studies conducted among older adults (aged ≥ 60 years) residing in India, reporting any risk factor for falls as exposure and unintentional fall as the outcome. MEDLINE, EMBASE, PsycInfo, CINAHL, and ProQuest Dissertations and Theses were searched until September 24, 2020. Where possible, data were synthesized using random‐effects meta‐analysis.

**Results:**

The literature search yielded 3445 records. Twenty‐two studies met the inclusion criteria of this systematic review, and 19 studies were included in the meta‐analysis. Out of the 22 included studies in the systematic review, 12 (out of 18) cross‐sectional studies, two case–control studies, and two cohort studies met more than 70% criteria in the respective Joanna Briggs Institute (JBI) checklists. Risk factors for falls among older adults in India included sociodemographic factors, environmental factors, lifestyle factors, physical and/or mental health conditions, and medical interventions.

**Conclusions:**

This systematic review and meta‐analysis provided a holistic picture of the problem in India by considering a range of risk factors such as sociodemographic, environmental, lifestyle, physical and/or mental health conditions and medical intervention. These findings could be used to develop falls prevention interventions for older adults in India.

**Systematic Review and Meta‐Analysis Registration:**

The systematic review and meta‐analysis protocol was registered with PROSPERO (registration number‐CRD42020204818).

## 1 INTRODUCTION

Falls are events that lead to a person coming to rest inadvertently at a lower level.^1^ Falls commonly occur in adults aged 60 years or more.^1^, ^2^ India is the second most populated country, and the number of older adults is estimated to be 137 million in 2021.^3^ The number of falls among older adults is increasing with the transition in demographics over time.^4^, ^5^ The pooled prevalence of falls among older adults in India is estimated to be 31% (95% confidence interval [CI]: 23%–39%).^6^

Falls can have a negative long‐term impact on the physical and psychological health and socioeconomic condition of the individual.^7^, ^8^, ^9^, ^10^, ^11^, ^12^, ^13^, ^14^, ^15^, ^16^, ^17^, ^18^ Impact on health includes morbidity and even mortality in severe cases.^7^, ^8^, ^9^, ^10^, ^11^, ^12^, ^13^, ^14^, ^15^, ^16^ Physical health consequences include injuries and fractures and reduced activities of daily living.^2^, ^7^, ^8^, ^11^ In India, every year, nearly 1.5–2 million older people suffer injuries due to falls, and 1 million succumb to death due to falls.^16^ Psychological health consequences include depression, anxiety, the fear of falling, and the lack of self‐confidence.^9^, ^11^, ^12^, ^13^, ^17^ Social consequences include the lack of social interaction leading to isolation.^9^ Economic consequences include increased health and social care costs.^18^ All these can take a toll on the overall quality of life.^9^, ^11^ Disability‐adjusted life years (DALYs) lost due to falls are also high.^15^

Several primary studies have been conducted in India to determine the risk factors for falls among older adults.^5^, ^19^, ^20^, ^21^, ^22^, ^23^, ^24^, ^25^ However, no systematic review has been conducted on this topic. Thus, the objective of this systematic review was to synthesize the existing evidence on the risk factors for falls among older adults in India. The intention was to provide a holistic picture of the problem in India by considering a range of risk factors such as sociodemographic, environmental, lifestyle, physical and/or mental health conditions, and medical intervention. These findings could be used to develop falls prevention interventions for older adults in India.

## 2 METHODS

The systematic review process adhered to the Joanna Briggs Institute (JBI) systematic reviews of etiology and risk guidelines^26^ and Preferred Reporting Items for Systematic Reviews and Meta‐Analyses (PRISMA).^27^ The review protocol was registered with PROSPERO (registration number: CRD42020204818). Two reviewers were involved in the process and independently screened the titles and abstracts and full texts of studies, assessed the methodological quality of studies, and extracted data from the studies (I. B. and B. A.). Any disagreements that arose between them were resolved through discussion. If consensus was not reached, a third reviewer was involved (K. C.).

## 3 INCLUSION CRITERIA

### 3.1 Population

The systematic review included studies conducted among older adults (aged ≥ 60 years) residing in India. A study was also eligible if the mean age of the participants was ≥60 years. Furthermore, if the study findings were stratified by age, required data were extracted from the relevant age group, that is, adults aged ≥60 years. If it was not possible to extract these findings, the study was excluded. Studies conducted in any setting, such as community, residential care, primary care, secondary care, and tertiary care, were eligible.

### 3.2 Exposure

Studies reporting any risk factors for falls as exposure were included.

### 3.3 Outcome

Studies reporting unintentional falls as outcomes were included (i.e., the actual occurrence of falls and not the risk or fear of falls). Studies reporting falls due to accidents or intentional actions like self‐harm or domestic violence were excluded.

### 3.4 Study design

Observational studies (cohort, case–control, and cross‐sectional studies) were included.

## 4 DATABASES AND SEARCH STRATEGY

We searched for a wide range of sources to find both published and unpublished studies. The following databases were searched for published studies: MEDLINE (Ovid; since 1946), EMBASE (Ovid; since 1974), PsycInfo (Ovid; since 1806), and CINAHL (EBSCOHost; since 1945), and the search for unpublished studies included ProQuest Dissertations and Theses. An initial limited search was carried out on MEDLINE and EMBASE databases using the keywords: “risk factors,” “falls,” and “India.” The titles and abstracts of the studies were screened for keywords, and the index terms used to describe the article were also identified. The search results were inspected to ensure that relevant articles were identified. Based on this, the search strategy for each database was developed in consultation with a senior research librarian and are detailed in the Supporting Information File: Appendix 1. All the databases were searched on September 24, 2020. No date or language restrictions were applied. The reference list of all the identified reviews and studies selected for inclusion in the systematic review were screened for additional studies.

## 5 STUDY SELECTION

Retrieved studies were collated and uploaded onto EndNote X9 (Clarivate Analytics), a reference management software.^28^ After the removal of duplicate studies, the titles, and abstracts of the remaining studies were screened for eligibility using the inclusion criteria. Studies identified as potentially eligible or those without an abstract had their full texts retrieved. Full texts of the studies were assessed for eligibility. Those that did not meet the inclusion criteria were excluded, and the reasons for exclusion are reported in the Supporting Information File: Appendix 2.

## 6 METHODOLOGICAL QUALITY ASSESSMENT

The included studies were critically assessed using the JBI checklists for observational studies.^26^, ^27^, ^29^ As recommended by JBI, a cut‐off score was not used to include/exclude studies. Hence, all studies irrespective of their methodological quality were included.

## 7 DATA EXTRACTION

Data were extracted from the included studies using a predeveloped and pretested data extraction, and we used Microsoft Word for this purpose. The following information was extracted: author and year of the study, name of the Indian state, study design, study period, study setting (e.g., community, primary care, secondary care, tertiary care), sample size, population characteristics (mean age [in years], number of females), risk factors explored, the definition of falls and assessment of falls (e.g., self‐reported/using medical notes or reports). Where possible, odds ratios (ORs) were extracted along with 95% CIs. Adjusted ORs were preferred over crude ORs. If only raw data were presented, ORs and 95% CIs were calculated. In case of missing or insufficient data in the paper, the corresponding author was emailed twice and requested to share the same.

## 8 DATA SYNTHESIS

All the studies were included in the narrative data synthesis. A meta‐analysis was conducted using Review Manager 5.4 (Cochrane Management System) if two or more studies reported the same or similar risk factors.^30^ Meta‐analysis was conducted separately for each study design. ORs were pooled together with 95% CIs using random‐effects meta‐analysis models. In the case of multiple categories in a study, two or more categories were combined to form a new category for analysis. For example, in some studies, socioeconomic status was reported as a higher class, middle class, and lower class. In this case, the lower and middle classes were combined to form one category and the higher class was considered as the reference group for the calculation of ORs. The standard errors were calculated using the following formula: standard error = (log upper CI–log lower CI)/3.92, in STATA 16 (Stata Corp.) for the creation of individual forest plots. The *I* ^2^ test was used to explore statistical heterogeneity across studies.

## 9 RESULTS

### 9.1 Study selection

**Figure 1:**
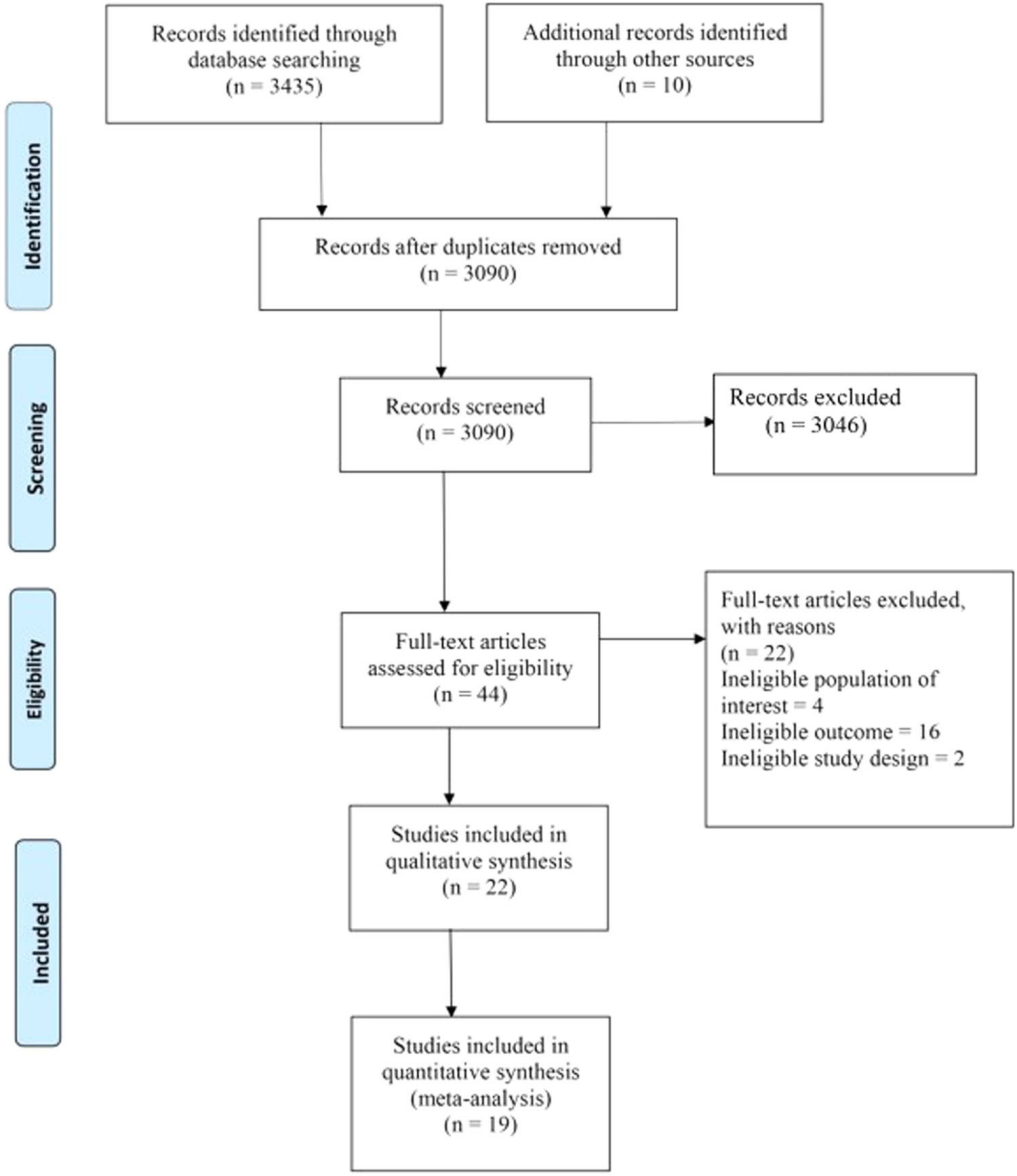
PRISMA flow diagram of the identification, screening, and eligibility of the included articles.

Figure 1 shows the PRISMA flow diagram of the identification, screening, and eligibility of included articles. Three thousand four hundred and forty‐five studies were identified, and after the removal of duplicates, 3090 studies were left for the title and abstract screening. After title and abstract screening, 44 studies were left for the full‐text screening. After the full‐text screening, 22 studies were included in this systematic review.^5^, ^19^, ^20^, ^21^, ^22^, ^23^, ^24^, ^25^, ^31^, ^32^, ^33^, ^34^, ^35^, ^36^, ^37^, ^38^, ^39^, ^40^, ^41^, ^42^, ^43^, ^44^ All the included studies were in the English language. Out of these 22 studies, 19 studies were included in the meta‐analysis.^5^, ^19^, ^23^, ^24^, ^25^, ^31^, ^32^, ^33^, ^34^, ^35^, ^36^, ^37^, ^38^, ^39^, ^40^, ^41^, ^42^, ^43^, ^44^

### 9.2 Characteristics of included studies

Characteristics of the included studies are presented in Table 1. Six studies were conducted in the northern states of India,^19^, ^20^, ^24^, ^31^, ^32^, ^41^ whereas 13 were conducted in the southern states.^5^, ^21^, ^22^, ^25^, ^33^, ^34^, ^36^, ^37^, ^39^, ^40^, ^42^, ^43^, ^44^ Only one study was conducted in an eastern state of India^38^ and another in a western state.^23^ One study was conducted in both western and southern states (Maharashtra and Karnataka, respectively).^35^ Eighteen studies were cross‐sectional,^19^, ^20^, ^21^, ^22^, ^23^, ^24^, ^25^, ^31^, ^32^, ^34^, ^35^, ^36^, ^37^, ^38^, ^39^, ^40^, ^41^, ^44^ two were case–control^5^, ^33^ and two were cohort.^42^, ^43^ The studies were conducted from 2001 onward. Seven studies were conducted in rural India^5^, ^22^, ^25^, ^31^, ^34^, ^40^, ^41^ and eight in urban.^21^, ^24^, ^33^, ^37^, ^38^, ^39^, ^43^, ^44^ Two studies were conducted both in rural and urban India^32^, ^42^ and five studies did not specify rural‐urban details.^19^, ^20^, ^23^, ^35^, ^36^ Sixteen studies were conducted in community care settings,^5^, ^20^, ^23^, ^24^, ^25^, ^31^, ^32^, ^34^, ^35^, ^37^, ^38^, ^39^, ^40^, ^41^, ^42^, ^44^ two were conducted in tertiary settings,^33^, ^43^ one in both community and tertiary settings^21^ and three in primary settings.^19^, ^22^, ^36^ The sample size ranged from 100 to 2049. The mean age ranged from 63.9 to 75.2 years. The studies included adults of both sexes except one which included only older women.^21^ The studies collected self‐reported data on exposures, and physical examinations were also conducted to assess the exposures in nine studies.^19^, ^25^, ^31^, ^32^, ^36^, ^37^, ^38^, ^39^, ^43^ Thirteen studies collected only self‐reported data on falls as reported by the patients/family members,^5^, ^20^, ^21^, ^22^, ^23^, ^24^, ^25^, ^33^, ^34^, ^36^, ^38^, ^39^, ^40^ eight studies used both self‐reported data and medical notes^19^, ^31^, ^32^, ^37^, ^41^, ^42^, ^43^, ^44^ and one study only used medical notes.^35^

**Table 1:** Characteristics of included studies

References R	Indian state	Study design	Study period	Study setting	Sample size (n)	Mean age (in years)	Females (n)	Risk factors explored	Definition of falls	Assessment of falls	Critical appraisal score (total % of “yes” to critical appraisal questions)
Johnson^21^	Kerala	Cross‐sectional	2002	Community and tertiary care	145	74.00	145	History of falls (S), area of injury in the body (S), location of falls (home/outside) (NS), required medical attention (S)	Not defined	Self‐reported by patients	38
Patil^37^	Karnataka	Cross‐sectional	2009–2010	Community care	416	Not reported	268	Medicine intake (S), alcohol consumption (S), smoking (S), physical activity (NS), usage of walking aid (S), usage of stairs (S), joint pains (NS), dizziness (S), diabetes (NS), balance (NS), gait (S), vision impairment (S), tremor (S), cataract (S), the urgency of micturition (NS), backache on walking (S), nonsteroidal anti‐inflammatory drugs (S), tricyclic antidepressants (S), usage of loose slippers outside the home (S)	Inadvertently coming to rest on the ground, floor, or other lower level excluding intentional change in position to rest on furniture, wall, or other objects	Self‐reported by patients and medical notes	100
Suryanarayana et al.^44^	Karnataka	Cross‐sectional	2010–2011	Community Care	416	67.00	268	Cluttering in the house (S), lighting inadequate (S), steps at the entrance of the house (S), the uneven floor of the house (S), split levels in the house (S), slippery floors of the house (S), inadequate handholds (NS), thresholds in the house (NS), carpets/loose rugs on the floor (NS), usage of Indian toilet (NS), uneven surfaces in the surroundings (NS)	Inadvertently coming to rest on the ground, floor, or other lower level excluding intentional change in position to rest on furniture, wall, or other objects	Self‐reported by patients and medical notes	63
Tripathy et al.^32^	Punjab	Cross‐sectional	2011–2012	Community Care	300	68.00	140	Age (NS), sex (NS), BMI (NS), balance (S), polypharmacy (S), residence place (urban/rural) (NS)	Not defined	Self‐reported by patients and medical notes	100
Dhargave and Sendhilkumar	Maharashtra Karnataka	Cross‐sectional	Not reported	Community care	163	74.61	87	Sex (S), vision impairment (S), medicine intake (S), usage of walking aid (S), vertigo (S), balance (S), gait (S), fear of fall (S), history of falls (S), acute medical problem (NS)	Inadvertently coming to rest on the ground, floor, or other lower level excluding intentional change in position to rest on furniture, wall, or other objects	Medical notes	75
Ravindran and Kutty^33^	Kerala	Case‐control	2013	Tertiary care	482 (includes both cases and controls)	69.31	286 (includes both cases and controls)	Age (S), history of falls (S), vision impairment (S), marital status (S), slippery floors (S)	Injurious falls were defined as falls that resulted in injuries that required hospitalization for at least 24 h	Self‐reported by patients	70
Saikia^38^	Assam	Cross‐sectional	2013	Community Care	400	Not reported	217	Age (S), gender (S), vision impairment (S), polypharmacy (S), functional status (S), gait (S), dementia (S)	Inadvertently coming to rest on the ground, floor, or other lower level, excluding intentional change in position to rest	Self‐reported by patients	50
Chacko and Thangaraj^34^	Tamil Nadu	Cross‐sectional	2015–2016	Community Care	655	Not reported	380	Age (S), sex (NS), functional disability (NS), formal education (NS), socioeconomic status (NS), arthritis (NS), diabetes (NS), hypertension (NS), vision impairment (NS), medicine intake (NS), alcohol consumption (NS), dizziness (S)	Coming to rest inadvertently on the ground or floor or other lower‐level occurring inside or outside the home	Self‐reported by patients and their family members	88
Rekha et al.^40^	Kerala	Cross‐sectional	2012–2013	Community care	202	69.50	110	Age (NS), sex (NS), formal education (S), marital status (NS), medicine intake (NS), fall history (S), existing morbidity (≥1) (S), multimorbidity (≥2) (NS)	An event that results in a person coming to rest inadvertently on the ground or floor or other levels	Self‐reported by patients	75
Sirohi et al.^31^	Haryana	Cross‐sectional	2015	Community care	456	69.40	256	Age (S), gender (S), socioeconomic status (S), urgency of micturition (S), diabetes (NS), hypertension (S), chronic respiratory morbidity (S), arthritis (S), functional disability (S), BMI (NS), balance (S), gait (S), vision impairment (S), hearing impairment (S), cognitive impairment (S), depression (S)	An event that results in a person coming to rest inadvertently on the ground or floor or other lower level	Self‐reported by patients and medical notes	100
Sharma et al.^25^	Telangana	Cross‐sectional	2012	Community care	561	67.50	281	Depression (S), BMI (S), cardiovascular disease (S)	A person was defined as a faller if s/he answered affirmatively to the following: “Have you fallen in the past 12 months?” and “If so, how many times?”	Self‐reported by patient	100
Balabaskaran and Dongre^22^	Pondicherry	Cross‐sectional	2017	Primary care	570	Not reported	Not reported	Type of house (Pucca, Kutcha, semi‐pucca) (NS), the flooring of the house (NS), flooring of the bathroom (S), type of house (NS), lighting in the living area and unstable furniture (NS), type of latrine (NS), flooring of the latrine (S), location of the latrine (NS)	Inadvertently coming to rest on the ground, floor, or other lower level, excluding intentional change in position to rest on furniture, wall, or other objects	Self‐reported by patients	38
Krishnaiah and Ramanathan^36^	Andhra Pradesh	Cross‐sectional	2016–2017	Primary care	382	63.90	202	Age (NS), gender (NS), formal education (NS), socioeconomic status (NS), cataract (S), systemic illness (S)	Unintentionally coming to the ground or some lower level and not as a result of a major intrinsic event (e.g., stroke) or overwhelming hazard	Self‐reported by patients	100
Pathania et al.^24^	Delhi	Cross‐sectional	2015	Community care	335	75.20	206	Age (S), sex (NS), existing morbidity (≥1) (S), formal education (NS), marital status (NS), pension (NS), usage of tobacco (S)	An event that resulted in a person coming to rest inadvertently on the ground or floor or other lower level	Self‐reported by patients	75
Adila^20^	Delhi	Cross‐sectional	Not reported	Community care	100	Not reported	54	Age (S), history of falls (S), vision impairment (S), polypharmacy (S), chronic disease (S), balance (S), vertigo (S), usage of walking aid (S)	Not defined	Self‐reported by patients	50
Peter et al.^5^	Tamil Nadu	Case–control	2013–2014	Community care	280 (includes both cases and controls)	66.00	151 (includes both cases and controls)	Physical activity (NS), vision impairment (NS), fear of falls (S), dizziness (S), diabetes (NS), alcohol consumption (NS), medicine intake (NS)	Inadvertently coming to rest on the ground, floor, or other lower level, excluding intentional change in position to rest in furniture, wall, or other objects	Self‐reported by patients	90
Jindal et al.^41^	Haryana	Cross‐sectional	2017	Community care	468	66.41	273	Gender (S), vertigo (S), hearing impairment (S), polypharmacy (S), slippery floors (S), weakness in any body part (S), joint pain (NS), chronic respiratory disease (S), hypertension (S), diabetes (NS), usage of stairs (S), functional disability (S), cognitive impairment (NS), vision impairment (NS), depression (S), ear discharge (NS), ear pain (NS), dim light (NS), uneven ground (NS), previous disability (S)	Inadvertently coming to rest on the ground, floor, or other lower level, excluding intentional change in position to rest on furniture, wall, or another object (fall within 1 year)	Self‐reported by patients and medical notes	100
Kumar and Ravindran^39^	Tamil Nadu	Cross‐sectional	2018	Community care	150	66.61	123	Age (NS), gender (NS), tremors (NS), multimorbidity (≥2) (NS), hypertension (NS), living alone (NS), diabetes (NS), vision impairment (NS), usage of walking aid (NS), joint pain (NS), physical activity (NS), BMI (NS), cataract (NS), balance (NS), gait (NS), forgetfulness (NS)	Inadvertently coming to rest on the ground, floor, or other lower level, excluding intentional change in position to rest in furniture, wall, or other objects	Self‐reported by patients	88
Pitchai et al.^23^	Maharashtra	Cross‐sectional	2016	Community care	2049	69.69	946	Age (S), gender (NS), formal education (S), marital status (S), living alone (S), socioeconomic status, living arrangement (NS), types of residency (community/institutional) (NS)	Any unintentional change in position where the person ends up on the floor, ground, or other lower level	Self‐reported by patients	63
Subramanian et al.^19^	Delhi	Cross‐sectional	2015–2017	Primary care	160	74.47	42	Fear of fall (S), pension (NS), formal education (NS), socioeconomic status (NS), alcohol consumption (NS), smoking (NS), diabetes (NS), joint pain (NS), the urgency of micturition (NS), chronic respiratory disease (NS), hypertension (NS), vision impairment (NS), functional disability (NS), anti‐anginal medications(S), opioids (S), self‐employment (S)	An event which results in a person coming to rest inadvertently on the ground floor or other lower level	Self‐reported by patients s and medical notes	100
Sasidharan et al.^42^	Kerala	Cohort	2015–2017	Community care	1000	72.70	568	Gender (S), movement disorders/Parkinson's disease (S), arthritis (S), functional disability (S), not the usage of hypertensive medications (S), living alone during daytime (S), history of falls (S), regular exercise or yoga (NS), age group (NS), diabetes (NS), hypertension (NS), asthma or COPD (NS), coronary artery disease (NS), cerebrovascular disease (S), alcohol consumption (NS), smoking (NS), knee pain (NS), numbness and paraesthesia of feet (S), urinary symptoms (S), vision impairment (NS)	Unintentionally coming to the ground or some lower level and other than as a consequence of sustaining a violent blow, loss of consciousness, sudden onset of paralysis as in stroke or an epileptic seizure	Self‐reported by patients and medical notes	100
Marmamula et al.^43^	Telangana	Cohort	2017–2019	Tertiary care	1074	74.40	686	Age (NS), gender (NS), hypertension (NS), diabetes (NS), hearing impairment (NS), depression (S), fear of falling (S), visual impairment (S)	Accidental coming to a halt at the level lower than their normal	Self‐reported by patients and medical notes	82

Abbreviations: NS, nonsignificant; S, significant (as reported by the study authors based on unadjusted/crude measures).

## 10 METHODOLOGICAL QUALITY OF INCLUDED STUDIES

The total critical appraisal scores for each study are presented in Table 1. Tables 2, 3, 4 report the detailed critical appraisal of the included studies.

**Table 2:** Critical appraisal results of cohort studies

Study	Q1	Q2	Q3	Q4	Q5	Q6	Q7	Q8	Q9	Q10	Q11	Total % of “yes” to critical appraisal questions
Sasidharan et al.^42^	Y	Y	Y	Y	Y	Y	Y	Y	Y	Y	Y	100 (11)
Marmamula et al.^43^	Y	Y	Y	Y	Y	Y	Y	Y	N	N	Y	82 (9)
Total % of “yes” to each critical appraisal question	100 (2)	100 (2)	100 (2)	100 (0)	100 (2)	100 (2)	100 (2)	100 (2)	50(1)	50 (1)	100 (2)	

Abbreviations: N, no; U, unclear; Y, yes.

1. Were the two groups similar and recruited from the same population?

2. Were the exposures measured similarly to assign people to both exposed and unexposed groups?

3. Was the exposure measured in a valid and reliable way?

4. Were confounding factors identified?

5. Were strategies to deal with confounding factors stated?

6. Were the groups/participants free of the outcome at the start of the study (or at the moment of exposure)?

7. Were the outcomes measured in a valid and reliable way?

8. Was the follow‐up time reported and sufficient to be long enough for outcomes to occur?

9. Was follow‐up complete, and if not, were the reasons to loss to follow‐up described and explored?

10. Were strategies to address incomplete follow‐up utilized?

11. Was appropriate statistical analysis used?

**Table 3:** Critical appraisal results of case–control studies

Study	Q1	Q2	Q3	Q4	Q5	Q6	Q7	Q8	Q9	Q10	Total % of “yes” to critical appraisal questions
Ravindran and Kutty^33^	U	N	Y	U	Y	Y	Y	Y	Y	Y	70 (7)
Peter et al.^5^	Y	Y	Y	U	Y	Y	Y	Y	Y	Y	90 (9)
Total % of “yes” to each critical appraisal question	50 (1)	50 (1)	100 (2)	0 (0)	100 (2)	100 (2)	100 (2)	100 (2)	100 (2)	100 (2)	

Abbreviations: N, no; U, unclear; Y, yes.

1. Were the groups comparable other than the presence of disease in cases or the absence of disease in controls?

2. Were cases and controls matched appropriately?

3. Were the same criteria used for the identification of cases and controls?

4. Was exposure measured in a standard, valid, and reliable way?

5. Was exposure measured in the same way for cases and controls?

6. Were confounding factors identified?

7. Were strategies to deal with confounding factors stated?

8. Were outcomes assessed in a standard, valid and reliable way for cases and controls?

9. Was the exposure period of interest long enough to be meaningful?

10. Was appropriate statistical analysis used?

**Table 4:** Critical appraisal results of cross‐sectional studies

Study	Q1	Q2	Q3	Q4	Q5	Q6	Q7	Q8	Total % of “yes” to critical appraisal questions
Johnson^21^	Y	Y	U	N	Y	N	N	N	38 (3)
Patil^37^	Y	Y	Y	Y	Y	Y	Y	Y	100 (8)
Suryanarayana et al.^44^	Y	Y	Y	U	U	N	Y	Y	63 (5)
Tripathy et al.^32^	Y	Y	Y	Y	Y	Y	Y	Y	100 (8)
Dhargave and Sendhilkumar^35^	Y	Y	Y	Y	N	N	Y	Y	75 (6)
Saikia^38^	Y	N	Y	Y	N	N	Y	N	50 (4)
Chacko and Thangaraj^34^	Y	Y	Y	N	Y	Y	Y	Y	88 (7)
Rekha et al.^40^	Y	Y	N	N	Y	Y	Y	Y	75 (6)
Sharma et al.^25^	Y	Y	Y	Y	Y	Y	Y	Y	100 (8)
Sirohi et al.^31^	Y	Y	Y	Y	Y	Y	Y	Y	100 (8)
Balabaskaran and Dongre^22^	N	Y	N	Y	N	N	Y	N	38 (3)
Krishnaiah and Ramanathan^36^	Y	Y	Y	Y	Y	Y	Y	Y	100 (8)
Pathania et al.^24^	Y	Y	N	U	Y	Y	Y	Y	75 (6)
Adila^20^	Y	Y	U	U	N	N	Y	Y	50 (4)
Jindal et al.^41^	Y	Y	Y	Y	Y	Y	Y	Y	100 (8)
Pitchai et al.^23^	Y	Y	Y	Y	N	N	Y	N	63 (5)
Kumar and Ravindran^39^	Y	Y	U	Y	Y	Y	Y	Y	88 (8)
Subramanian et al.^19^	Y	Y	Y	Y	Y	Y	Y	Y	100 (8)
Total % of “yes” to each critical appraisal question	94 (16)	94 (16)	65 (11)	71 (12)	71 (12)	65 (11)	94 (16)	76 (13)	

Abbreviations: N, no; U, unclear; Y, yes.

1. Were the criteria for inclusion in the sample clearly defined?

2. Were the study subjects and the setting described in detail?

3. Was the exposure measured in a valid and reliable way?

4. Were objective, standard criteria used for measurement of the condition?

5. Were confounding factors identified?

6. Were strategies to deal with confounding factors stated?

7. Were the outcomes measured in a valid and reliable way?

8. Was appropriate statistical analysis used?

Two cohort studies attained more than 70% JBI criteria, that is, answered affirmatively to at least eight questions on the checklist.^42^, ^43^ The two groups for comparison were similar in characteristics and recruited from the same population in both the studies.^42^, ^43^ Measurement of exposures was done in a valid and reliable way and clearly described in both the studies.^42^, ^43^ Both the studies identified confounding factors and used multiple logistic regression analysis to deal with confounding.^42^, ^43^ The patients were free of the outcome (i.e., no falls) before inclusion in the studies and used standard definitions of falls.^42^, ^43^ The follow‐up time was at least 1 year which was sufficient to assess falls.^42^, ^43^ In one study, there was no information on the follow‐up of patients, and the strategies to address incomplete follow‐up were also not described.^43^ Appropriate statistical analysis was used as both the studies utilized regression analysis.^42^, ^43^

Both the case–control studies attained more than 70% JBI criteria, that is, answered affirmatively to at least seven questions on the checklist.^5^, ^33^ Cases and controls were not matched appropriately in one study.^33^ For each of the studies, the same criteria were used for the identification of cases and controls.^5^, ^33^ It was unclear if the validity of exposure measurement was done in a standard, valid and reliable way.^5^, ^33^ However, measurement of exposure was done using the same method for cases and controls.^5^, ^33^ Both the studies identified confounders and used multivariable logistic regression analysis to deal with the potential confounding variables. Standard definitions of falls were used to assess falls in a standard, valid and reliable way for both cases and controls.^5^, ^33^ The exposure period of interest was at least 6 months in both the studies, which was enough to assess falls. Appropriate statistical analyses were used as multivariable regression analyses were conducted in both the studies.^5^, ^33^

Twelve out of 18 cross‐sectional studies included in the systematic review attained more than 70% JBI criteria, that is, answered affirmatively to at least six questions on the checklist.^19^, ^24^, ^25^, ^31^, ^32^, ^34^, ^35^, ^36^, ^37^, ^39^, ^40^, ^41^ All the studies reported inclusion criteria except one^22^ and study settings and patients except one.^38^ The measurement of exposure was unclear in three studies^20^, ^21^, ^39^ and was not described in three studies.^22^, ^24^, ^40^ All the studies defined falls succinctly except three.^21^, ^34^, ^40^ Five studies did not identify the confounders and strategies to deal with the same.^20^, ^22^, ^23^, ^35^, ^38^ However, studies that mentioned confounders reported age and sex as the most common confounders. In the four studies with insufficient statistical analyses, multivariable logistic regression could have been conducted.^21^, ^22^, ^23^, ^38^

## 11 META‐ANALYSIS

**Figure 2:**
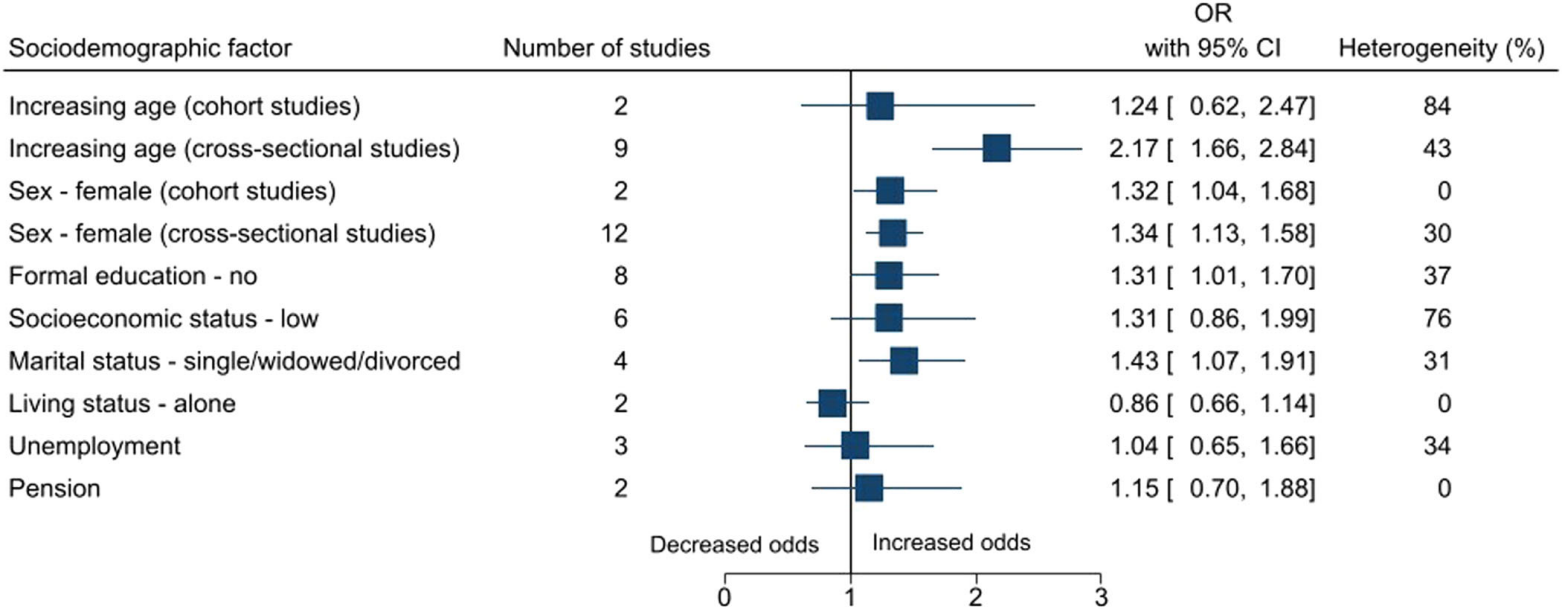
Summary forest plot of the association between sociodemographic factors and falls.

**Figure 3:**
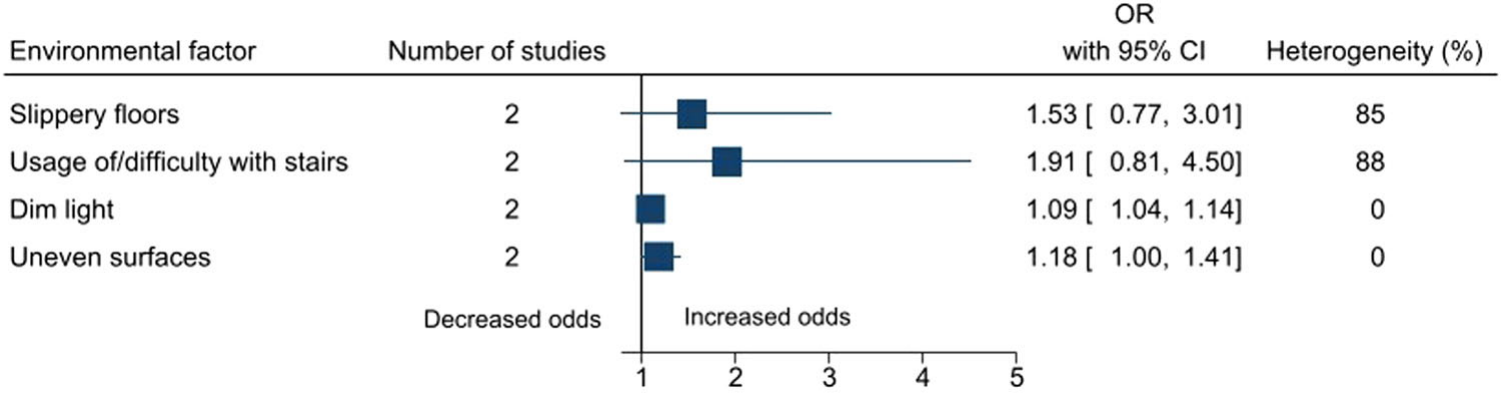
Summary forest plot of the association between environmental factors and falls.

**Figure 4:**
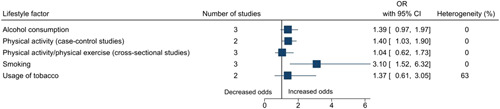
Summary forest plot of the association between lifestyle factors and falls.

**Figure 5:**
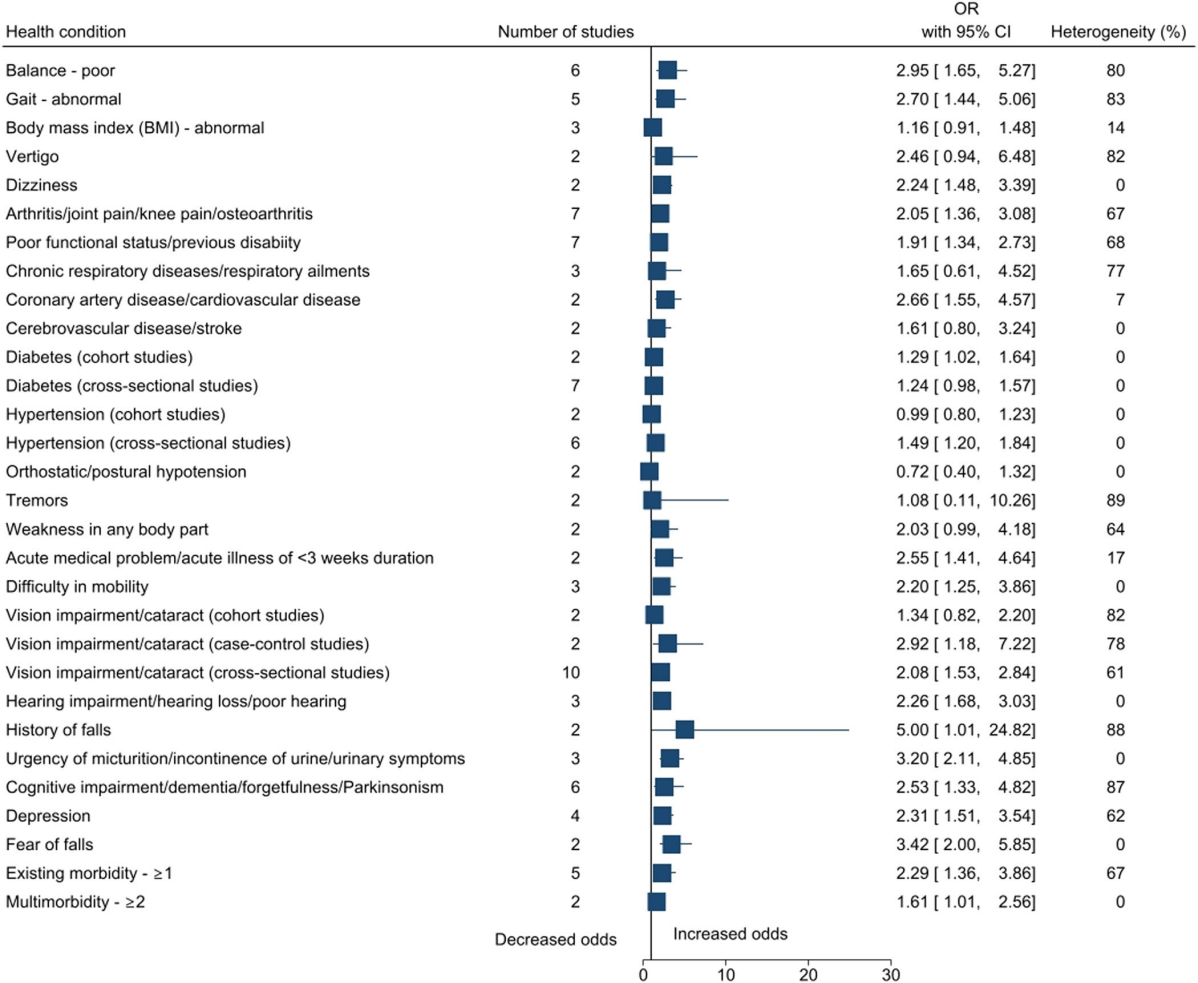
Summary forest plot of the association between physical and/or mental health conditions and falls.

**Figure 6:**
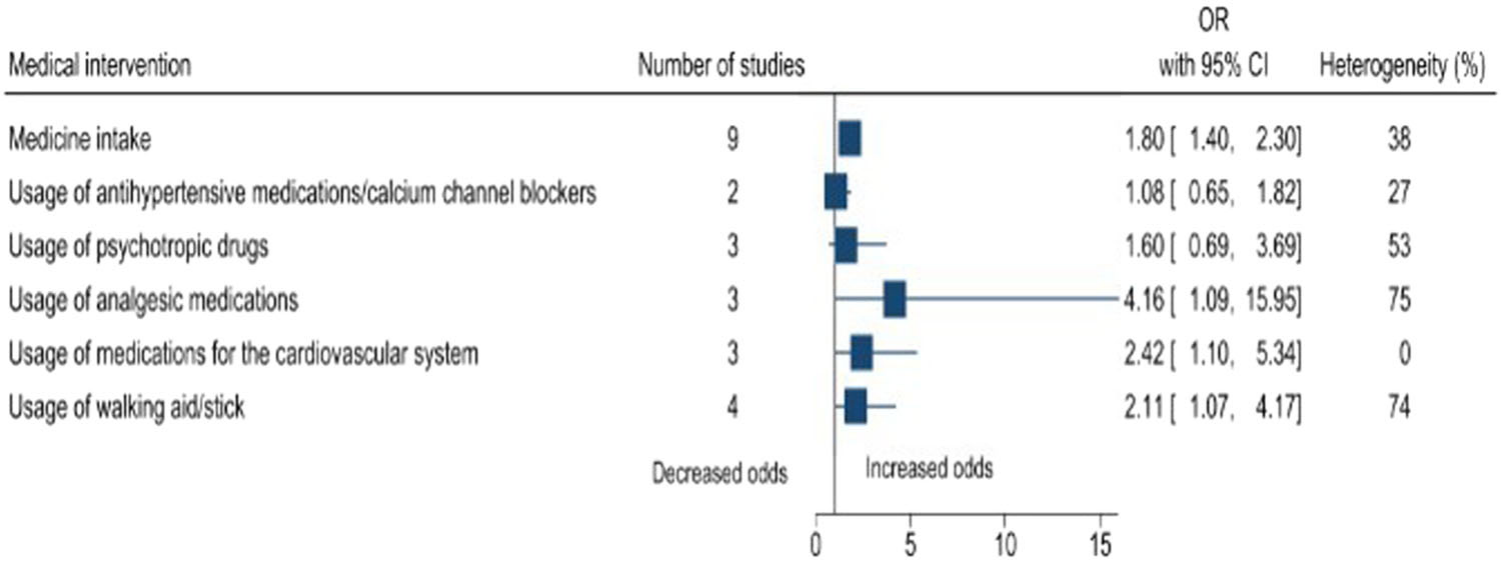
Summary forest plot of the association between medical interventions and falls.

Statistically significant risk factors for falls among older adults in India included sociodemographic factors: increasing age (OR: 2.17, 95% CI: 1.66–2.84), female sex (cohort studies: 1.32, 1.04–1.68; case–control studies: 1.34, 1.13–1.58), no formal education (1.31, 1.01–1.70), and marital status—single/widowed/divorced (1.43, 1.07–1.91); an environmental factor: dim light (1.09, 1.04–1.14); lifestyle factors: physical activity (1.40, 1.03–1.90) and smoking (3.10, 1.52–6.32); physical and/or mental health conditions: poor balance (2.95, 1.65–5.27), abnormal gait (2.70, 1.44–5.06), dizziness (2.24, 1.48–3.39), arthritis/joint pain/knee pain/osteoarthritis (2.05, 1.36–3.08), functional status/previous disability (1.91, 1.34–2.73), coronary artery disease/cardiovascular disease (2.66, 1.55–4.57), diabetes (1.29, 1.02–1.64), hypertension (1.49, 1.20–1.84), difficulty in mobility (2.20, 1.25–3.86), vision impairment/cataract (case–control studies: 2.92, 1.18–7.22; cross‐sectional studies: 2.08, 1.53–2.84), hearing impairment/hearing loss/poor hearing (2.26, 1.68–3.03), history of falls (5.00, 1.01–24.82), urgency of micturition/incontinence of urine/urinary symptoms (3.20, 2.11–4.85), cognitive impairment/dementia/forgetfulness/Parkinsonism (2.53, 1.33–4.82), depression (2.31, 1.51–3.54), fear of falls (3.42, 2.00–5.85), acute medical problem/acute illness of <3 weeks duration (2.55, 1.41–4.64), existing morbidity ≥1 (2.29, 1.36 to 3.86) and multimorbidity ≥2 (1.61, 1.01 to 2.56); medical interventions: medicine intake (1.80, 1.40–2.30), usage of analgesic medications (4.16, 1.09–15.95), usage of medications for the cardiovascular system (2.42, 1.10–5.34), and usage of walking aid/stick (2.11, 1.07–4.17). The summary forest plots for the broad categories of risk factors included in the meta‐analysis are shown in Figures 2, 3, 4, 5, 6. The individual forest plots are represented in the Supporting Information File: Appendix 3 to Figures [Link], [Link], [Link], [Link], [Link]. Supporting Information File: Appendix 4—Figures [Link], [Link], [Link], [Link] show the summary forest plots for the broad categories of risk factors that could not be included in the meta‐analysis.

## 12 DISCUSSION

Risk factors for falls among older adults in India included sociodemographic factors, environmental factors, lifestyle factors, physical and/or mental health conditions, and medical interventions. Some of the review findings were consistent with previous systematic reviews conducted worldwide including increasing age,^45^, ^46^, ^47^, ^48^ female sex,^49^ dim light,^50^ poor balance,^38^, ^51^, ^52^ abnormal gait,^38^, ^51^, ^52^ dizziness,^53^, ^54^ poor functional status,^45^, ^55^, ^56^ hearing impairment/hearing loss,^57^, ^58^ cerebrovascular disease/stroke,^58^ arthritis/joint pain,^39^, ^59^, ^60^ urgency of micturition/incontinence of urine/urinary symptoms,^58^, ^61^, ^62^, ^63^ vision impairment,^56^, ^64^, ^65^, ^66^ diabetes,^67^, ^68^, ^69^ hypertension,^70^ difficulty in mobility,^58^, ^71^ history of falls,^38^, ^71^ depression,^45^, ^72^ dementia,^56^ cognitive impairment,^38^, ^45^, ^56^, ^72^ fear of falls,^73^, ^74^ multimorbidity,^55^, ^75^ medicine intake,^71^, ^76^, ^77^, ^78^, ^79^, ^80^, ^81^, ^82^ usage of medications for the cardiovascular system,^76^, ^77^, ^82^ and usage of walking aid/stick.^83^ This review also highlighted some additional risk factors for falls among older adults. For example, sociodemographic factors such as being single/widowed/divorced and no formal education, lifestyle factors such as physical activity and smoking, and physical and/or mental health conditions such as acute medical problem/acute illness of <3 weeks duration and existing morbidity (≥1) and medical interventions such as usage of analgesic medications. Globally, age is a well‐known risk factor for falls.^45^, ^46^, ^47^, ^48^ In this review, age was found to be a significant risk factor in the meta‐analysis conducted for cross‐sectional studies, however, not for cohort studies. In terms of the hierarchy of study designs, cohort studies are considered better than cross‐sectional studies. However, in this case, there were only two cohort studies, and the statistical heterogeneity was high (84%). On the other hand, there were nine cross‐sectional studies, and the statistical heterogeneity was 43%. It should also be noted that we included only those studies that focused on older adults, and the age range was already narrow. In this review, physical activity was found to be a risk factor. Intuitively, one would expect the opposite, and this issue requires further investigation. The possible reason could be not following the recommended physical activity guidelines, quantity or quality wise.^84^

In total, three studies could not be included in the meta‐analysis. In two studies, it was not possible to estimate the ORs due to insufficient raw data, however, other relevant information was extracted.^20^, ^21^ Another study mentioned unique risk factors which were not reported in any other study.^22^ In addition, there were some unique risk factors in the other 19 studies that could not be included in the meta‐analysis. More primary research needs to be conducted on several risk factors for which meta‐analysis could not be performed. The included studies were mostly conducted in the northern and southern states of India, and thus, primary studies need to be conducted in other parts of the country for a more complete picture. The majority of the included studies used the standard definitions of falls. However, the information on falls and risk factors were mostly self‐reported by the patients or their family members. Therefore, future research studies should also incorporate other ways in data collection to minimize the risk of recall bias, such as using medical notes and reports and doing physical examinations. Some of the included studies had poor response rates, and the exact reason should be explored and addressed. For example, the way people are approached to participate in a study. Some of the included studies did not adjust for confounders, and this should be addressed in future research studies.

To the best of our knowledge, this was the first systematic review to synthesize the existing evidence on the risk factors for falls among older adults in India. A robust process was followed using JBI and PRISMA guidelines. The probability of missing relevant articles was minimal as we searched for both published and unpublished studies, without any date or language restrictions, and a large number of studies were included. Although the definition of each risk factor was not provided in the articles, in the meta‐analysis, the reviewers tried their best to pool together risk factors having the same or similar meaning. The sample size of the included studies ranged from 100 to 2049, and one might question how reliable would the pooled estimates be when dealing with such a diverse set of samples. To explain this, a sensitivity analysis could have been done by excluding smaller studies, but the problem was to determine how small was small and where to draw the line. Also, the diverse sampling techniques could affect the reliability of the findings.

The systematic review findings could be valid in neighboring South Asian nations because of similarities in population characteristics, sociocultural setups, and healthcare systems. For example, similar to the findings of our review, a primary study conducted in Pakistan reported the association between diabetes and falls among older adults,^85^ and research shows that South Asians are more likely to have diabetes.^86^ Hence, the findings could be used by a range of stakeholders (including policymakers) in the South Asian region to develop falls prevention targeted interventions, depending on the exact risk factor. If there is more than one risk factor, a multifactorial intervention is recommended to prevent falls.^87^, ^88^ It should be noted that the “one‐size‐fits‐all” concept should not be applied, and “need‐sensitive” interventions are required. One such example could be yoga‐based interventions.^89^

## 13 CONCLUSION

This systematic review and meta‐analysis reported a wide range of risk factors for falls among older adults in India such as sociodemographic, environmental, lifestyle, physical and/or mental health condition, and medical intervention. These findings could be used to develop fall prevention interventions for older adults in India.

## AUTHOR CONTRIBUTIONS

**Isha Biswas**: Conceptualization; data curation; formal analysis; investigation; methodology; resources; software; validation; visualization; writing—original draft; writing—review and editing. **Busola Adebusoye**: Data curation; formal analysis; investigation; methodology; software; validation; writing—review and editing. **Kaushik Chattopadhyay**: Conceptualization; funding acquisition; investigation; methodology; resources; software; supervision; validation; visualization; writing—original draft; writing—review and editing.

## CONFLICTS OF INTEREST

The authors declare no conflicts of interest.

## TRANSPARENCY STATEMENT

The manuscript is an honest, accurate, and transparent account of the study being reported; that no important aspects of the study have been omitted; and that any discrepancies from the study as planned (and, if relevant, registered) have been explained.

## Supporting information

Supporting information.

Supporting information.

Supporting information.

Supporting information.

Supporting information.

Supporting information.

Supporting information.

Supporting information.

Supporting information.

Supporting information.

Supporting information.

Supporting information.

Supporting information.

Supporting information.

Supporting information.

Supporting information.

Supporting information.

Supporting information.

Supporting information.

Supporting information.

Supporting information.

Supporting information.

Supporting information.

Supporting information.

Supporting information.

Supporting information.

Supporting information.

Supporting information.

Supporting information.

Supporting information.

Supporting information.

Supporting information.

Supporting information.

Supporting information.

Supporting information.

Supporting information.

Supporting information.

Supporting information.

Supporting information.

Supporting information.

Supporting information.

Supporting information.

Supporting information.

Supporting information.

Supporting information.

Supporting information.

Supporting information.

Supporting information.

Supporting information.

Supporting information.

Supporting information.

Supporting information.

Supporting information.

Supporting information.

Supporting information.

Supporting information.

Supporting information.

Supporting information.

Supporting information.

Supporting information.

## Data Availability

The authors confirm that the data supporting the findings of this study are available within the article [and/or] its supplementary materials.
