# Supplementary figures and images for: Risk factors for falls among older adults in India: A systematic review and meta‐analysis
Source: Health Sci Rep. 2022 Jun 21;5(4):e637. doi: 10.1002/hsr2.637 (PMC9213836; doi:10.1002/hsr2.637)

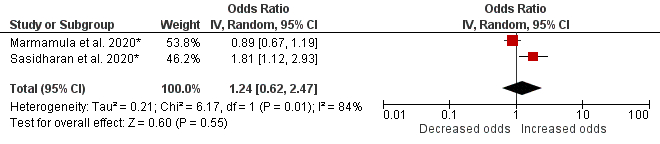

Supplement: Supplementary file 1 — Supporting information. [file HSR2-5-e637-s027.jpg]

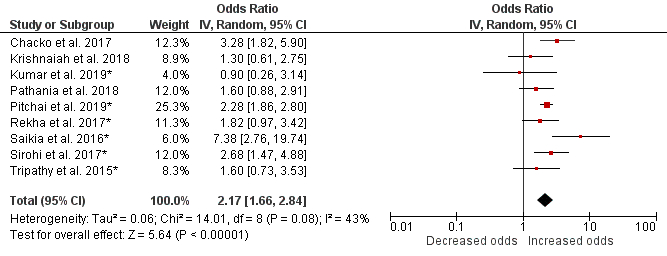

Supplement: Supplementary file 2 — Supporting information. [file HSR2-5-e637-s012.jpg]

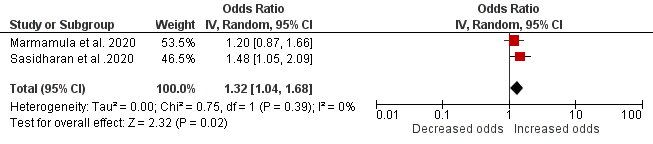

Supplement: Supplementary file 3 — Supporting information. [file HSR2-5-e637-s039.jpg]

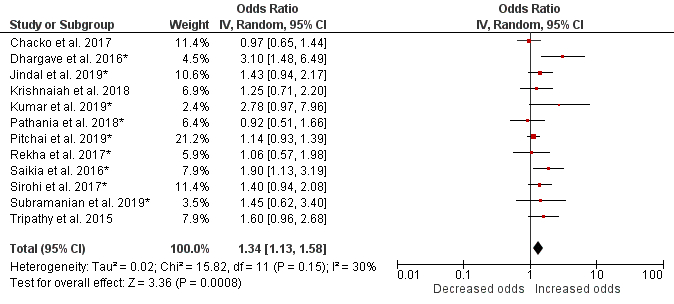

Supplement: Supplementary file 4 — Supporting information. [file HSR2-5-e637-s033.jpg]

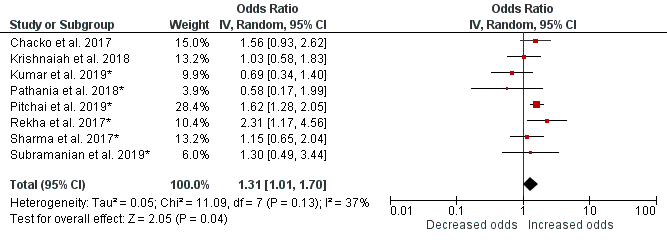

Supplement: Supplementary file 5 — Supporting information. [file HSR2-5-e637-s005.jpg]

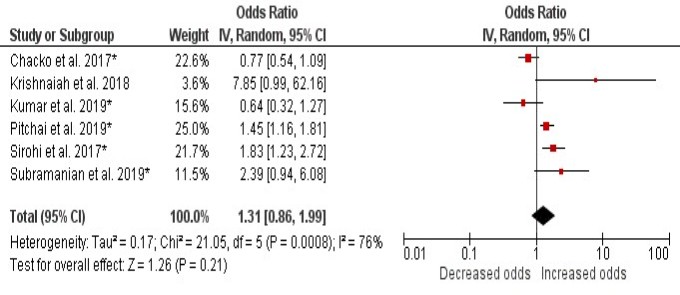

Supplement: Supplementary file 6 — Supporting information. [file HSR2-5-e637-s021.jpg]

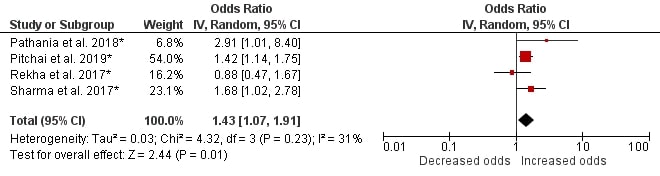

Supplement: Supplementary file 7 — Supporting information. [file HSR2-5-e637-s052.jpg]

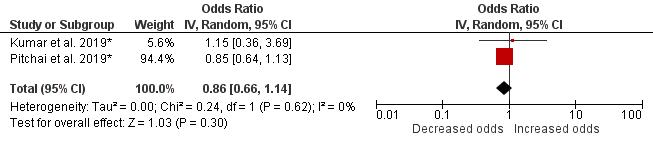

Supplement: Supplementary file 8 — Supporting information. [file HSR2-5-e637-s003.jpg]

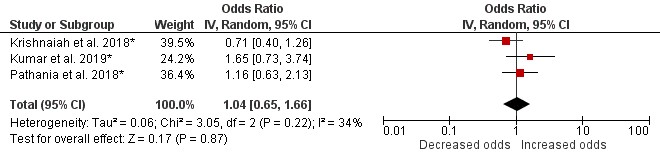

Supplement: Supplementary file 9 — Supporting information. [file HSR2-5-e637-s036.jpg]

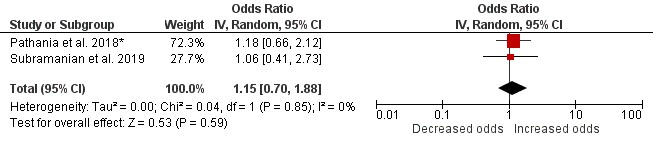

Supplement: Supplementary file 10 — Supporting information. [file HSR2-5-e637-s022.jpg]

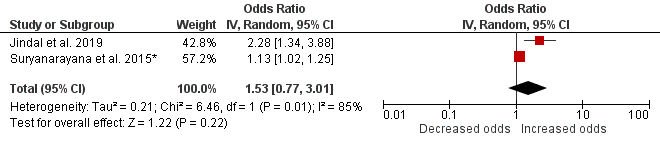

Supplement: Supplementary file 11 — Supporting information. [file HSR2-5-e637-s049.jpg]

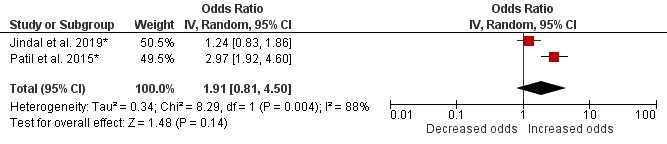

Supplement: Supplementary file 12 — Supporting information. [file HSR2-5-e637-s040.jpg]

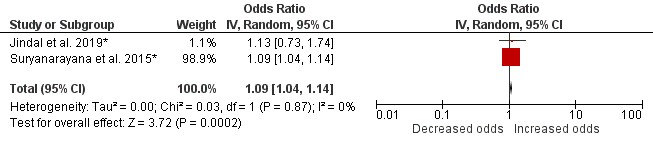

Supplement: Supplementary file 13 — Supporting information. [file HSR2-5-e637-s014.jpg]

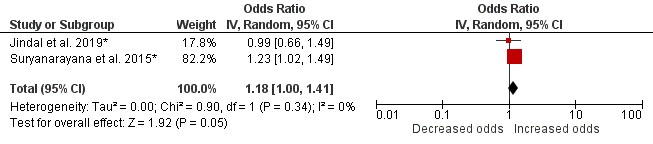

Supplement: Supplementary file 14 — Supporting information. [file HSR2-5-e637-s030.jpg]

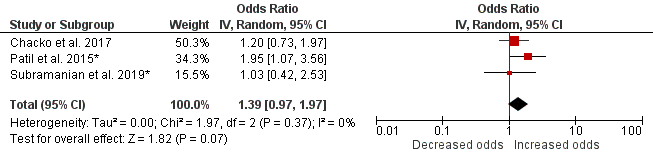

Supplement: Supplementary file 15 — Supporting information. [file HSR2-5-e637-s056.jpg]

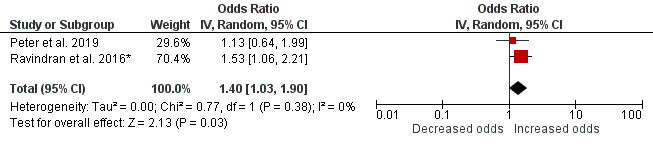

Supplement: Supplementary file 16 — Supporting information. [file HSR2-5-e637-s032.jpg]

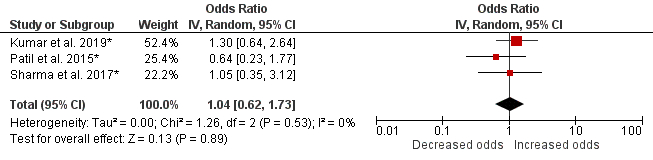

Supplement: Supplementary file 17 — Supporting information. [file HSR2-5-e637-s007.jpg]

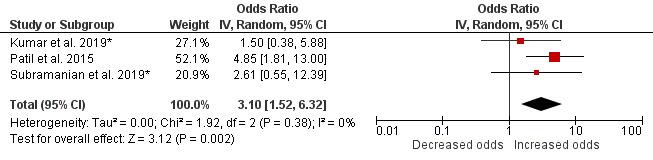

Supplement: Supplementary file 18 — Supporting information. [file HSR2-5-e637-s058.jpeg]

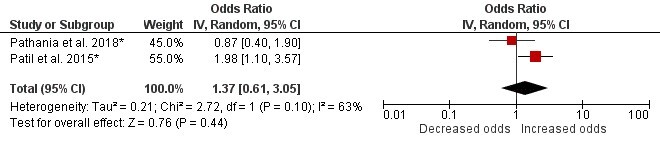

Supplement: Supplementary file 19 — Supporting information. [file HSR2-5-e637-s045.jpeg]

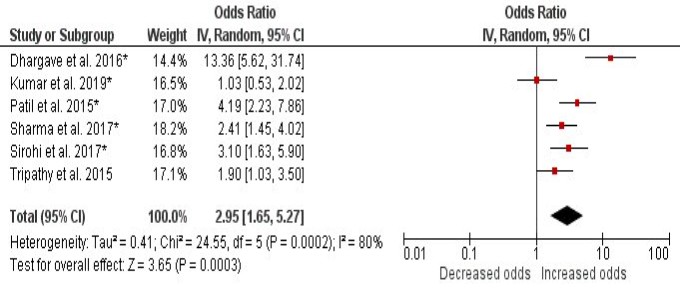

Supplement: Supplementary file 20 — Supporting information. [file HSR2-5-e637-s034.jpg]

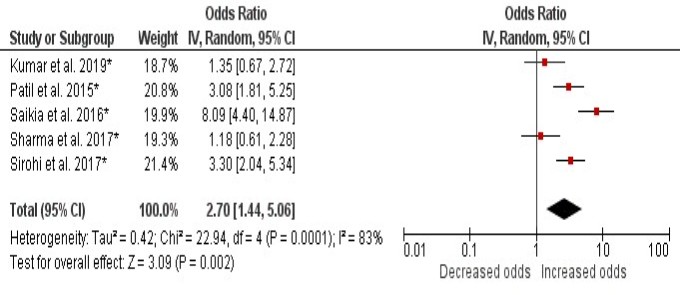

Supplement: Supplementary file 21 — Supporting information. [file HSR2-5-e637-s009.jpg]

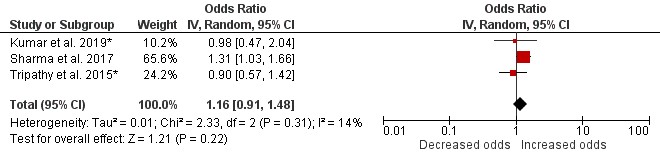

Supplement: Supplementary file 22 — Supporting information. [file HSR2-5-e637-s028.jpg]

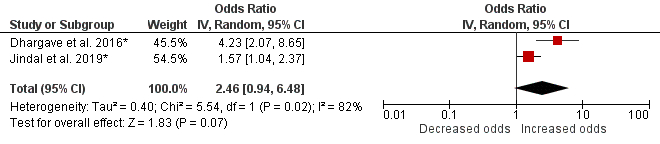

Supplement: Supplementary file 23 — Supporting information. [file HSR2-5-e637-s055.jpg]

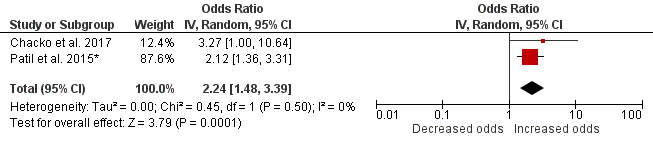

Supplement: Supplementary file 24 — Supporting information. [file HSR2-5-e637-s041.jpg]

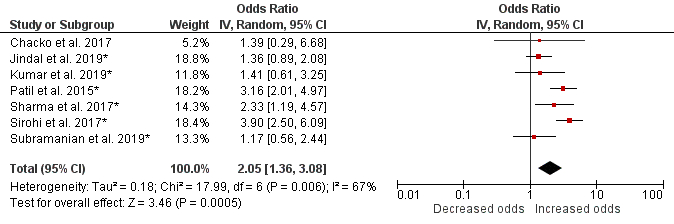

Supplement: Supplementary file 25 — Supporting information. [file HSR2-5-e637-s016.jpg]

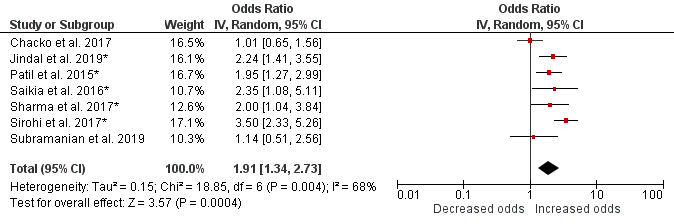

Supplement: Supplementary file 26 — Supporting information. [file HSR2-5-e637-s019.jpg]

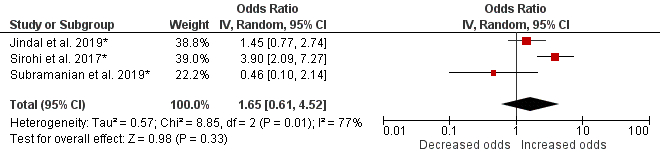

Supplement: Supplementary file 27 — Supporting information. [file HSR2-5-e637-s048.jpg]

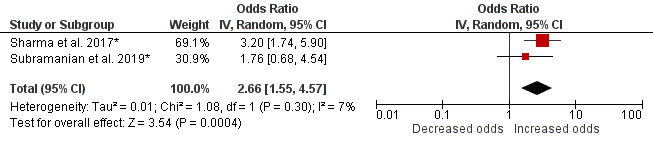

Supplement: Supplementary file 28 — Supporting information. [file HSR2-5-e637-s050.jpg]

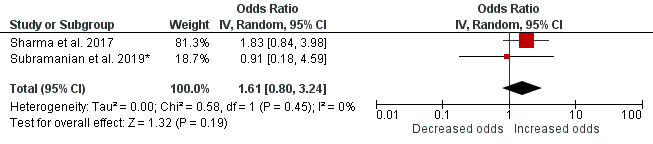

Supplement: Supplementary file 29 — Supporting information. [file HSR2-5-e637-s023.jpg]

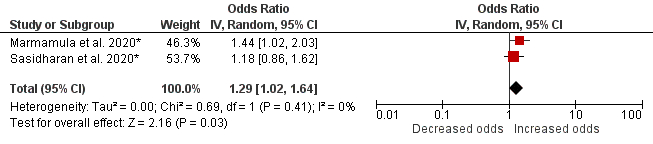

Supplement: Supplementary file 30 — Supporting information. [file HSR2-5-e637-s047.jpg]

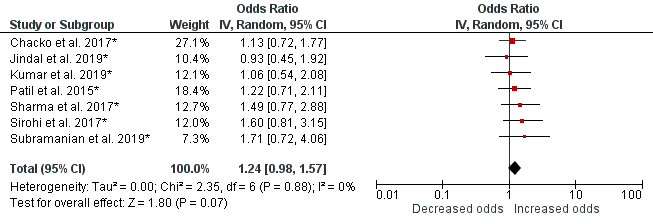

Supplement: Supplementary file 31 — Supporting information. [file HSR2-5-e637-s020.jpg]

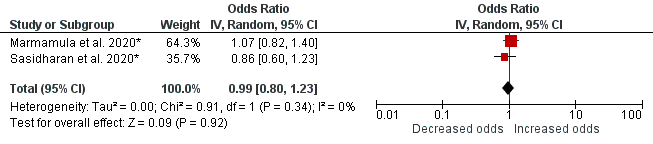

Supplement: Supplementary file 32 — Supporting information. [file HSR2-5-e637-s015.jpg]

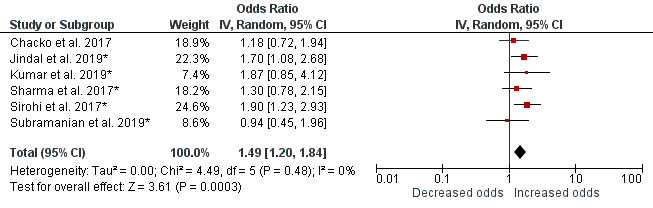

Supplement: Supplementary file 33 — Supporting information. [file HSR2-5-e637-s042.jpg]

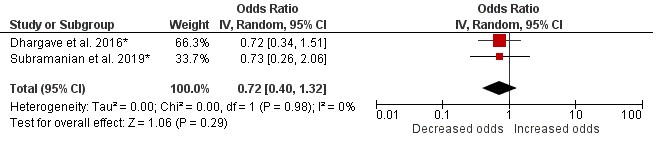

Supplement: Supplementary file 34 — Supporting information. [file HSR2-5-e637-s054.jpg]

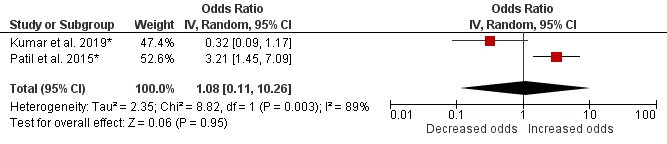

Supplement: Supplementary file 35 — Supporting information. [file HSR2-5-e637-s029.jpg]

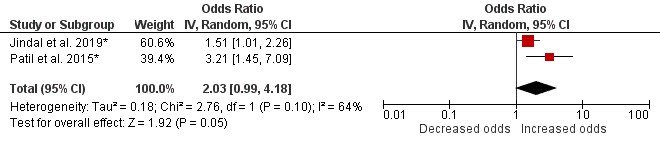

Supplement: Supplementary file 36 — Supporting information. [file HSR2-5-e637-s008.jpg]

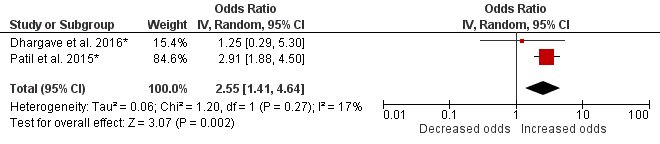

Supplement: Supplementary file 37 — Supporting information. [file HSR2-5-e637-s035.jpg]

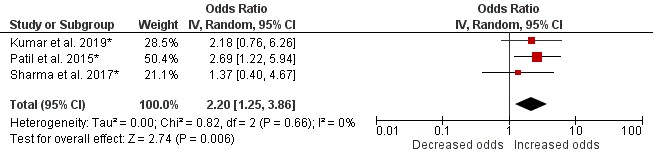

Supplement: Supplementary file 38 — Supporting information. [file HSR2-5-e637-s031.jpg]

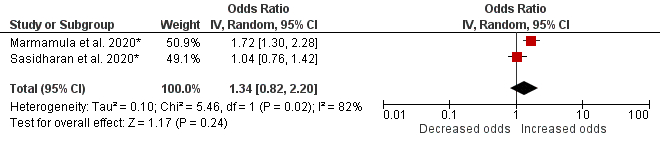

Supplement: Supplementary file 39 — Supporting information. [file HSR2-5-e637-s006.jpg]

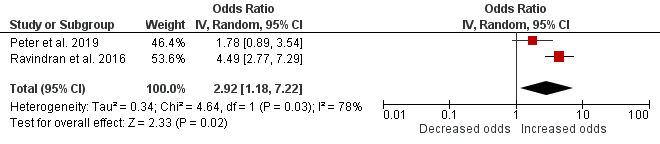

Supplement: Supplementary file 40 — Supporting information. [file HSR2-5-e637-s059.jpg]

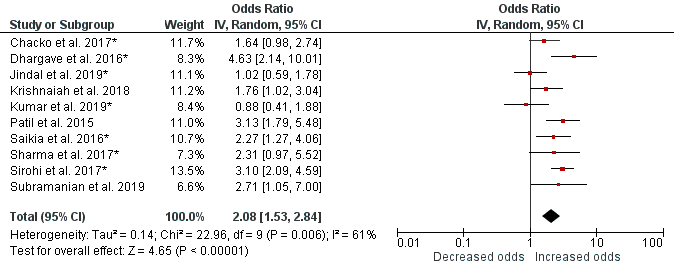

Supplement: Supplementary file 41 — Supporting information. [file HSR2-5-e637-s026.jpg]

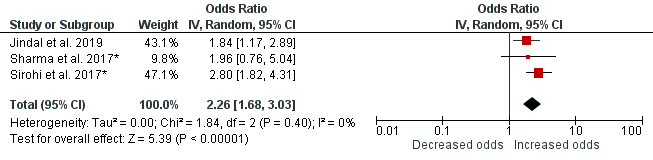

Supplement: Supplementary file 42 — Supporting information. [file HSR2-5-e637-s001.jpg]

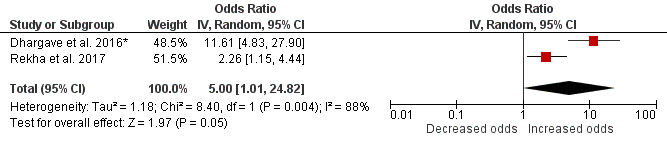

Supplement: Supplementary file 43 — Supporting information. [file HSR2-5-e637-s038.jpg]

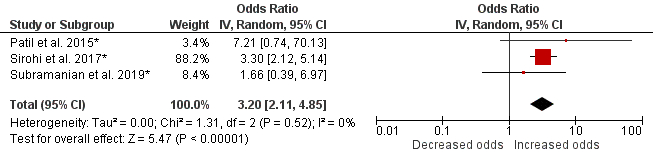

Supplement: Supplementary file 44 — Supporting information. [file HSR2-5-e637-s051.jpg]

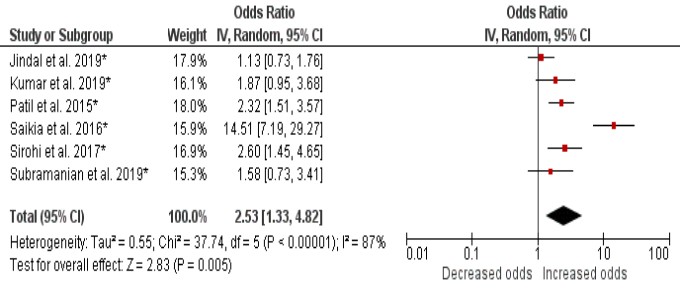

Supplement: Supplementary file 45 — Supporting information. [file HSR2-5-e637-s018.jpg]

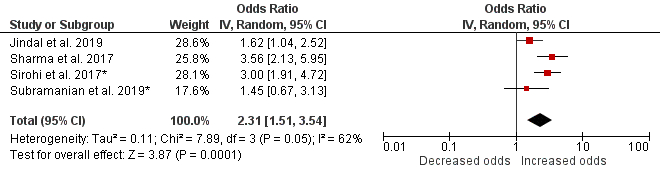

Supplement: Supplementary file 46 — Supporting information. [file HSR2-5-e637-s010.jpg]

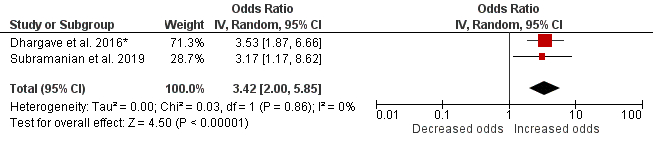

Supplement: Supplementary file 47 — Supporting information. [file HSR2-5-e637-s044.jpg]

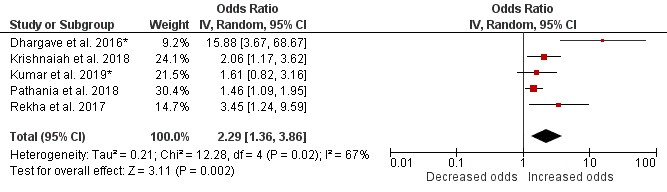

Supplement: Supplementary file 48 — Supporting information. [file HSR2-5-e637-s046.jpg]

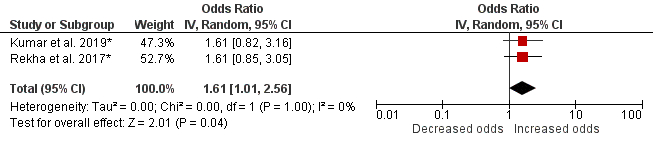

Supplement: Supplementary file 49 — Supporting information. [file HSR2-5-e637-s013.jpg]

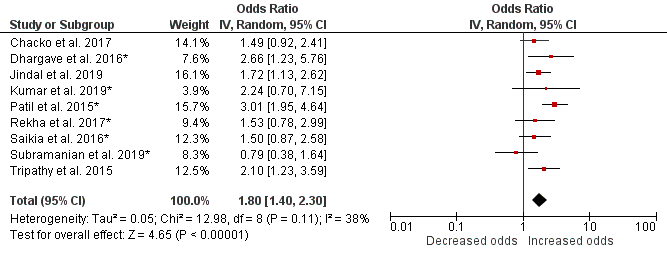

Supplement: Supplementary file 50 — Supporting information. [file HSR2-5-e637-s043.jpg]

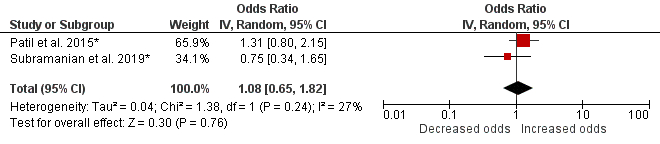

Supplement: Supplementary file 51 — Supporting information. [file HSR2-5-e637-s011.jpg]

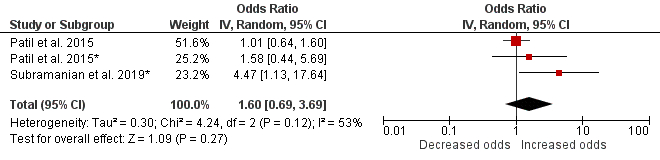

Supplement: Supplementary file 52 — Supporting information. [file HSR2-5-e637-s017.jpg]

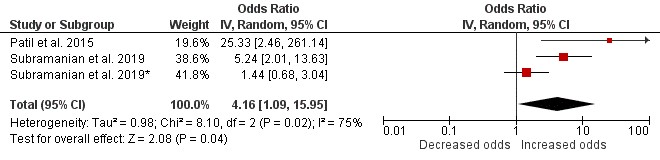

Supplement: Supplementary file 53 — Supporting information. [file HSR2-5-e637-s053.jpg]

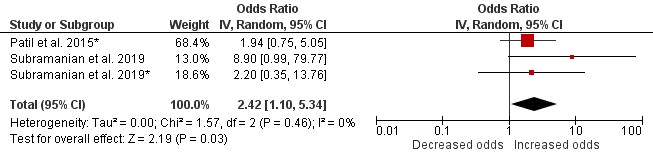

Supplement: Supplementary file 54 — Supporting information. [file HSR2-5-e637-s037.jpg]

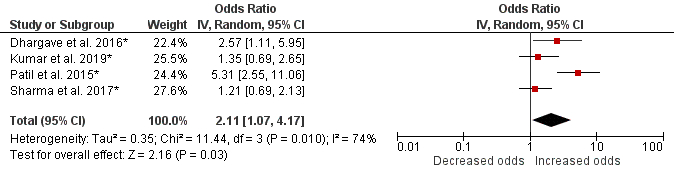

Supplement: Supplementary file 55 — Supporting information. [file HSR2-5-e637-s002.jpg]

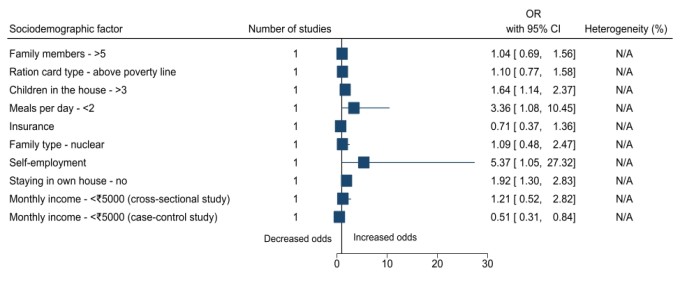

Supplement: Supplementary file 56 — Supporting information. [file HSR2-5-e637-s025.jpg]

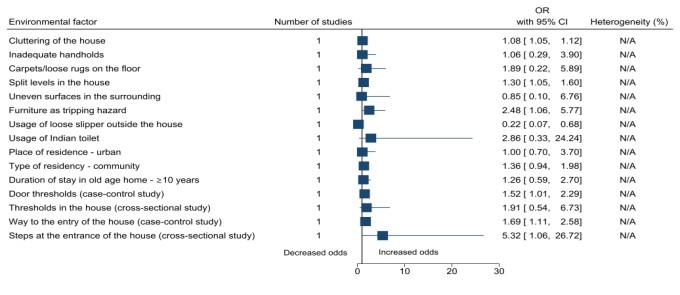

Supplement: Supplementary file 57 — Supporting information. [file HSR2-5-e637-s060.jpg]

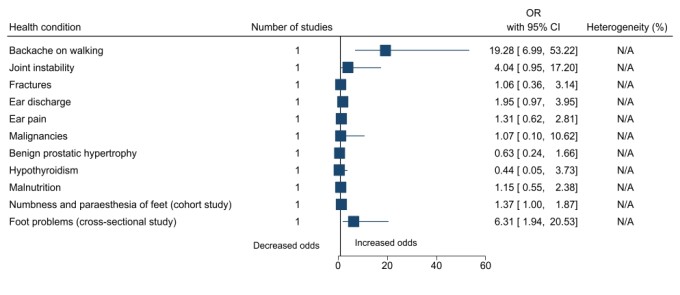

Supplement: Supplementary file 58 — Supporting information. [file HSR2-5-e637-s057.jpg]

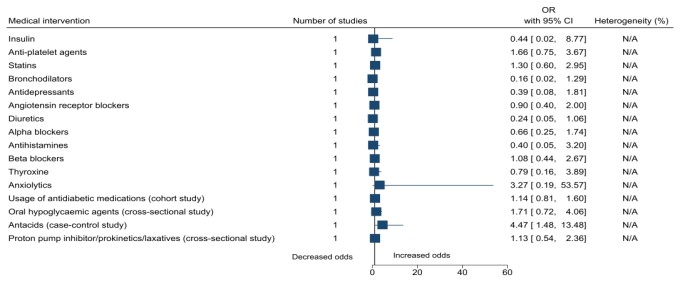

Supplement: Supplementary file 59 — Supporting information. [file HSR2-5-e637-s024.jpg]
