# Supplementary material for: Risk factors for falls among older adults in India: A systematic review and meta‐analysis
Source: Health Sci Rep. 2022 Jun 21;5(4):e637. doi: 10.1002/hsr2.637 (PMC9213836; doi:10.1002/hsr2.637)
Supplement: Supplementary file 60 — Supporting information. [file HSR2-5-e637-s004.docx]

**Online supplementary file**

**Appendix 1 - Search strategies.**

1. **MEDLINE (Ovid); Date of search: 24^th^ September 2020; 635 records.**
2. exp Risk factors/
3. exp Risk/
4. Risk*.mp
5. exp Risk assessment/
6. Predict*.mp
7. Characteristics*.mp
8. Exp Human characteristics/
9. Factor*.mp
10. Determinant*.mp
11. Epidemiolog*.mp
12. exp Epidemiology/
13. Odds ratio*.mp
14. Risk ratio*.mp
15. exp Accidental falls/
16. Accidental fall*.mp
17. Fall injur*.mp
18. Fell.mp
19. Slip*.mp
20. Trip*.mp
21. Stumble.mp
22. exp Gait/
23. exp Postural balance/
24. exp Adult/
25. Old* adult*.mp
26. exp Aged/
27. exp Aging/
28. exp India/
29. India*.mp
30. Bharat*.mp
31. Hindustan*.mp
32. 1 or 2 or 3 or 4 or 5 or 6 or 7 or 8 or 9 or 10 or 11 or 12 or 13
33. 14 or 15 or 16 or 17 or 18 or 19 or 20 or 21 or 22
34. 23 or 24 or 25 or 26
35. 27 or 28 or 29 or 30
36. 31 and 32 and 33 and 34

**B. EMBASE (Ovid); Date of search: 24^th^ September 2020; 2072 records.**

1. exp Risk factor/
2. exp Fall risk/
3. exp Risk/
4. exp Risk assessment/
5. exp Fall risk assessment/
6. Risk*.mp.
7. Predict*.mp.
8. exp Human characteristic/
9. Characteristic*.mp
10. Factor*.mp.
11. Determinant*.mp.
12. Epidemiolog*.mp.
13. exp Epidemiology/
14. exp Odds ratio/
15. Risk ratio*.mp.
16. Fall*.mp.
17. Accidental fall*.mp.
18. exp falling/
19. Fell. mp.
20. Slip*.mp.
21. Fall injur*.mp.
22. Trip*.mp.
23. Stumble.mp.
24. exp collapse/
25. Collapse*.mp.
26. exp adult/
27. Old* adult*.mp.
28. exp aged/
29. exp aging/
30. exp India/
31. India*.mp.
32. Bharat*.mp.
33. Hindustan*.mp.
34. 1 or 2 or 3 or 4 or 5 or 6 or 7 or 8 or 9 or 10 or 11 or 12 or 13 or 14 or 15
35. 16 or 17 or 18 or 19 or 20 or 21 or 22 or 23 or 24 or 25
36. 26 or 27 or 28 or 29
37. 30 or 31 or 32 or 33
38. 34 and 35 and 36 and 37
39. **PsycInfo (Ovid); Date of search: 24^th^ September 2020; 117 records.**
40. exp Risk factors/
41. Risk*.mp
42. exp Risk assessment/
43. Predictor*.mp
44. Characteristic*.mp
45. Human characteristic*.mp
46. Factor*.mp
47. Determinant*.mp
48. exp Epidemiology/
49. Epidemiolog*.mp
50. Odds ratio*.mp
51. Risk ratio*.mp
52. exp Falls/
53. Falls*.mp
54. Accidental fall*.mp
55. Fell.mp
56. exp Injuries/
57. Slip*.mp
58. Trip*.mp
59. Stumble.mp
60. exp Gait/
61. Postural balance.mp
62. Collapse*.mp
63. Adult*.mp
64. Old* adult*.mp
65. exp Aging/
66. Aged.mp
67. India*.mp
68. Bharat*.mp
69. Hindustan*.mp
70. 1or 2 or 3 or 4 or 5 or 6 or 7 or 8 or 9 or 10 or 11 or 12
71. 13 or 14 or 15 or 16 or 17 or 18 or 19 or 20 or 21 or 22 or 23
72. 24 or 25 or 26 or 27
73. 28 or 29 or 30
74. 31 and 32 and 33 and 34

**D.** **CINAHL (EBSCOHost); Date of search: 24^th^ September 2020; 162 records.**

S1 (MH "Risk factors+")

S2 "Risk*"

S3 (MH "Risk assessment")

S4 "Predictor*"

S5 "Determinant*"

S6 (MH "Epidemiology")

S7 (MH "Odds ratio")

S8 "Risk ratio*"

S9 (MH "Accidental falls")

S10 "Fall*"

S11 "Fall injur*"

S12 "Slip*"

S13 "Trip*"

S14 "Stumble"

S15 (MH "Gait+")

S16 (MH "Adult+")

S17 (MH "India")

S18 ((MH "India")) AND (S1 OR S2 OR S3 OR S4 OR S5 OR S6 OR S7 OR S8)

S19 (((MH "India")) AND (S1 OR S2 OR S3 OR S4 OR S5 OR S6 OR S7 OR S8)) AND (S9 OR S10 OR S11 OR S12 OR S13 OR S14 OR S15)

S20 ((((MH "India")) AND (S1 OR S2 OR S3 OR S4 OR S5 OR S6 OR S7 OR S8)) AND (S9 OR S10 OR S11 OR S12 OR S13 OR S14 OR S15)) AND (S16 AND S19)

**E. ProQuest Dissertations and Theses (ProQuest Central); Date of search: 24^th^ September 2020; 449 records.**

[(Risk OR Risk factor* OR Predictor* OR Determinant*) AND (Fall* or Slip* or Trip* or Stumble*) AND (Adult*)](https://search-proquest-com.ezproxy.nottingham.ac.uk/myresearch/savedsearches.checkdbssearchlink:rerunsearch/1867018/SavedSearches?site=pqdt&t:ac=SavedSearches) AND (India*)

**Appendix 2 - Reasons for exclusion of full-text studies.**

**Ineligible population of interest: 4**

1. Dandona R, Kumar GA, Ivers R, et al. Characteristics of non-fatal fall injuries in rural India. *Injury Prevention*. 2010;16(3):166-71.

2. Kavita R, Girish N, Gururaj G, et al. Burden, characteristics, and outcome of injury among females: observations from Bengaluru, India. *Women's Health Issues.* 2011;21(4):320-6.

3. Gosavi SV, Deshmukh PR. Epidemiology of injuries in rural Wardha, central India. *Medical Journal Armed Forces India*. 2014;70(4):380-2.

4. Jagnoor J, Suraweera W, Keay L, et al. Childhood and adult mortality from unintentional falls in India. *Bulletin World Health Organization*. 2011;89(10):733-40.

**Ineligible outcome: 16**

1**.** Ahuja K, Sen S, Dhanwal D, et al. Risk factors and epidemiological profile of hip fractures in Indian population: a case-control study. *Osteoporosis Sarcopenia.* 2017;(3):138-48.

2. Dhibar D, Gogate Y, Aggarwal S, et al. Predictors and outcome of fragility hip fracture: a prospective study from north India. *Indian Journal of Endocrinology and Metabolism.* 2019;23(3):282-88.

3. Rashid I, Tiwari P, Lehl S, et al. Prevalence and determinants of fall risk among elderly inpatients at a tertiary care public teaching hospital. *Journal of Gerontology and Geriatrics*. 2018; 67:96-102.

4. Kendhapedi KK, Devasenapathy N. Prevalence and factors associated with frailty among community-dwelling older people in rural Thanjavur district of south India: a cross-sectional study. *BMJ Open*. 2019;9(10): e032904.

5. Kalsait AS, Lakshmiprabha R, Sraswati I, et al. Correlation of cognitive impairment with functional mobility and risk of fall in elderly individuals. *Indian Journal of Physiotherapy and Occupational Therapy*. 2017;11(2):7-11.

6. Cardona M, Joshi R, Ivers RQ, et al. The burden of fatal and non-fatal injury in rural India. *Injury Prevention*. 2008;14(4):232-37.

7. Kalaiselvan G, Dongre AR, Mahalakshmy T, et al. Epidemiology of injury in rural Pondicherry, India. *Journal of Injury and Violence Research*. 2011;3(2):62-7.

8. Dash SK, Panigrahi R, Palo N, et al. Fragility hip fractures in elderly patients in Bhubaneswar, India (2012-2014): a prospective multicenter study of 1031 elderly patients. *Geriatric Orthopaedic Surgery and Rehabilitation*. 2015;6(1):11-15.

9. Agarwal P, Upadhyay P, Raja K, et al. A demographic profile of traumatic and non-traumatic spinal injury cases: a hospital-based study from India. *Spinal Cord.* 2007;45(9):597-602.

10. Kaur M, Kaur J, Devgun P, et al. Post fall health consequences among elderly. *IOSR Journal of Nursing and Health Science (IOSR-JNHS).*2018;7(5):34-7.

11. Joshi K, Kumar R, Avasthi A, et al. Morbidity profile and its relationship with disability and psychological distress among elderly people in northern India. *International Journal of Epidemiology.* 2003;32(6):978-87.

12. Lugade VA. Balance control and stability during gait: an evaluation of fall risk among elderly adults. *University of Oregon Dissertations*. 2011.

13. Mane AB, Sanjana T, Patil PR, et al. Prevalence and correlates of fear of falling among elderly population in urban area of Karnataka, India. *Journal of Mid-life Health*. 2014;5(3):150-5.

14. Kulkarni S, Gadkari R, Nagarkar A, et al. Risk factors for fear of falling in older adults in India. *Journal of Public Health (Germany).* 2020;28(2):123-29.

15. Joseph A, Bagavandas M. Non-fatal home injuries among the elderly in Tamil Nadu, India. *Indian Journal of Community Medicine.* 2019;44(1):81-4.

16. Aras RY, Narayan V, D'Souza ND, et al. Assessment of accident risk among elderly in domestic environment: a cross-sectional study in rural south Karnataka, India. *Annals of Tropical Medicine and Public Health*. 2012;5(6):565-68.

**Ineligible study design: 2**

1. Joseph A, Kumar D, Bagavandas M, et al. A review of epidemiology of fall among elderly in India. *Indian Journal of Community Medicine*. 2019;44(2):166-68.

2. Jagnoor J, Keay L, Ganguli A, et al. Fall related injuries: a retrospective medical review study in north India. *Injury.* 2012;43(12):1996-2000.

**Appendix 3 - Individual forest plots of the association between risk factors and falls.**

**Figure S1:** Forest plot of the association between age (cohort studies) and falls. (*Unadjusted; The authors’ definition of higher age was used which varied from ≥70 to ≥80 years. In case of multiple categories in a study, 2 or more categories were combined to form a new category. Lower age was the reference group).

**Figure S2:** Forest plot of the association between age (cross-sectional studies) and falls. (*Unadjusted; The authors’ definition of higher age was used which varied from ≥70 to ≥80 years. In case of multiple categories in a study, 2 or more categories were combined to form a new category. Lower age was the reference group).

**Figure S3:** Forest plot of the association between sex (cohort studies) and falls. (*Unadjusted; Studies reporting gender and sex as risk factors were combined as "sex" to obtain a pooled estimate. Men were the reference group)

**Figure S4:** Forest plot of the association between sex (cross-sectional studies) and falls. (*Unadjusted; Studies reporting gender and sex as risk factors were combined as "sex" to obtain a pooled estimate. Men were the reference group)

**Figure S5:** Forest plot of the association between formal education and falls. (*Unadjusted; The authors’ definition of no formal education was used. In case of multiple categories in a study, 2 or more categories were combined to form a new category. Formal education was the reference group).

**Figure S6:** Forest plot of the association between socioeconomic status and falls. (*Unadjusted; The authors’ definition of low socioeconomic status was used. In case of multiple categories in a study, 2 or more categories were combined to form a new category. High socioeconomic status was the reference group).

**Figure S7:** Forest plot of the association between marital status and falls. (*Unadjusted; Marital status – married was the reference group).

**Figure S8:** Forest plot of the association between living status and falls. (*Unadjusted; Living with others (family or relatives) was the reference group).

**Figure S9:** Forest plot of the association between unemployment and falls. (*Unadjusted; No unemployment was the reference group).

**Figure S10:** Forest plot of the association between pension and falls. (*Unadjusted; No pension was the reference group).

**Figure S11:** Forest plot of the association between slippery floors and falls. Forest plot of the association between slippery floors and falls (*Unadjusted; No slippery floor was the reference group).

**Figure S12:** Forest plot of the association between usage of/difficulty with stairs and falls. (*Unadjusted; No usage of/no difficulty with stairs was the reference group).

**Figure S13:** Forest plot of the association between dim light and falls. (*Unadjusted; No dim light was the reference group).

**Figure S14:** Forest plot of the association between uneven surfaces and falls. (*Unadjusted; No uneven surface was the reference group).

**Figure S15:** Forest plot of the association between alcohol consumption and falls. (*Unadjusted; No alcohol consumption was the reference group).

**Figure S16:** Forest plot of the association between physical activity (case-control studies) and falls. (*Unadjusted; No physical activity/exercise/yoga was the reference group).

**Figure S17:** Forest plot of the association between physical activity/physical exercise (cross-sectional studies) and falls. (*Unadjusted; No physical activity/exercise/yoga was the reference group).

**Figure S18:** Forest plot of the association between smoking and falls. (*Unadjusted; No smoking was the reference group).

**Figure S19:** Forest plot of the association between usage of tobacco and falls. (*Unadjusted; No usage of tobacco was the reference group).

**Figure S20:** Forest plot of the association between balance and falls. (*Unadjusted; The authors’ definition of poor balance was used. Adequate balance was the reference group).

**Figure S21:** Forest plot of the association between gait and falls. (*Unadjusted; The authors’ definition of abnormal gait was used. Normal gait was the reference group).

**Figure S22:** Forest plot of the association between Body Mass Index (BMI) and falls. (*Unadjusted; The authors’ definition of abnormal BMI was used. Standard BMI definition was used. Normal BMI was the reference group. Sharma et al assessed BMI as a continuous measure).

**Figure S23:** Forest plot of the association between vertigo and falls. (*Unadjusted; No vertigo was the reference group).

**Figure S24:** Forest plot of the association between dizziness and falls. (*Unadjusted; No dizziness was the reference group).

**Figure S25:** Forest plot of the association between arthritis/joint pain/knee pain/osteoarthritis and falls. (*Unadjusted; The authors’ definition of arthritis/ joint pain/ knee pain was used. No arthritis/ joint pain/ knee pain was the reference group).

**Figure S26:** Forest plot of the association between functional status/previous disability and falls. (*Unadjusted; The authors’ definition of functional disability was used. No functional disability was the reference group).

**Figure S27:** Forest plot of the association between chronic respiratory disease/respiratory ailments and falls. (*Unadjusted; The authors’ definition of chronic respiratory diseases/respiratory ailments was used. No chronic respiratory diseases/respiratory ailments were the reference group).

**Figure S28:** Forest plot of the association between coronary artery disease/cardiovascular disease and falls. (*Unadjusted; The authors’ definition of coronary artery disease/cardiovascular disease was used. No coronary artery disease/cardiovascular disease was the reference group).

**Figure S29:** Forest plot of the association between cerebrovascular disease/stroke and falls. (*Unadjusted; The authors’ definition of cerebrovascular disease/stroke was used. No cerebrovascular disease/stroke was the reference group).

**Figure S30:** Forest plot of the association between diabetes (cohort studies) and falls. (*Unadjusted; The authors’ definition of diabetes was used. No diabetes was the reference group).

**Figure S31:** Forest plot of the association between diabetes (cross-sectional studies) and falls. (*Unadjusted; The authors’ definition of diabetes was used. No diabetes was the reference group).

**Figure S32:** Forest plot of the association between hypertension (cohort studies) and falls. (*Unadjusted; No hypertension was the reference group).

**Figure S33:** Forest plot of the association between hypertension (cross-sectional studies) and falls. (*Unadjusted; No hypertension was the reference group).

**Figure S34:** Forest plot of the association between orthostatic/postural hypotension and falls. (*Unadjusted; No orthostatic/postural hypotension was the reference group).

**Figure S35:** Forest plot of the association between tremors and falls. (*Unadjusted; No tremor was the reference group).

**Figure S36:** Forest plot of the association between weakness in any body part and falls. (*Unadjusted; The authors’ definition of weakness in any body part was used. No weakness in any body part was the reference group).

**Figure S37:** Forest plot of the association between acute medical problem/acute illness of <3 weeks duration and falls. (*Unadjusted; The authors’ definition of acute medical problem/acute illness of <3 weeks duration was used. No acute medical problem/acute illness of <3 weeks duration was the reference group).

**Figure S38:** Forest plot of the association between difficulty in mobility and falls. (*Unadjusted; The authors’ definition of difficulty in mobility was used. No difficulty in mobility was the reference group).

**Figure S39:** Forest plot of the association between vision impairment/cataract (cohort studies) and falls. (*Unadjusted; The authors’ definition of vision impairment/cataract was used. No vision impairment/cataract was the reference group).

**Figure S40:** Forest plot of the association between vision impairment/cataract (case-control studies) and falls. (*Unadjusted; The authors’ definition of vision impairment/cataract was used. No vision impairment/cataract was the reference group).

**Figure S41:** Forest plot of the association between vision impairment (cross-sectional studies) and falls. (*Unadjusted; The authors’ definition of vision impairment/cataract was used. No vision impairment/cataract was the reference group).

**Figure S42:** Forest plot of the association between hearing impairment/hearing loss/poor hearing and falls. (*Unadjusted; The authors’ definition of hearing impairment was used. No hearing impairment was the reference group).

**Figure S43:** Forest plot of the association between history of falls and falls. (*Unadjusted; The authors’ definition of history of falls was used. No history of falls was the reference group).

**Figure S44:** Forest plot of the association between urgency of micturition/incontinence of urine/urinary symptoms and falls. (*Unadjusted; No urgency of micturition/ incontinence of urine was the reference group).

**Figure S45:** Forest plot of the association between cognitive impairment/dementia/forgetfulness/Parkinsonism and falls. (*Unadjusted; The authors’ definition of cognitive impairment/dementia/forgetfulness/Parkinsonism was used. No cognitive impairment was the reference group).

**Figure S46:** Forest plot of the association between depression and falls. (*Unadjusted; No depression was the reference group).

**Figure S47:** Forest plot of the association between fear of falls and falls. (*Unadjusted; No fear of falls was the reference group).

**Figure S48:** Forest plot of the association between existing morbidity - ≥1 and falls. (*Unadjusted; The authors’ definition of existing morbidity was used. No morbidity was the reference group).

**Figure S49:** Forest plot of the association between multimorbidity - ≥2 and falls. (*Unadjusted; The authors’ definition of multimorbidity was used which varied from ≥2 morbidities. No multimorbidity was the reference group).

**Figure S50:** Forest plot of the association between medicine intake and falls. (*Unadjusted; The authors’ definition of medicine intake was used. No medicine intake was the reference group).

**Figure S51:** Forest plot of the association between usage of antihypertensive medications/calcium channel blockers and falls. (*Unadjusted; No usage of anti-hypertensive medications was the reference group).

**Figure S52:** Forest plot of the association between usage of psychotropic drugs and falls. (*Unadjusted; No usage of psychotropic drugs was the reference group).

**Figure S53:** Forest plot of the association between usage of analgesic medications and falls. (*Unadjusted; No usage of analgesic medications was the reference group).

**Figure S54:** Forest plot of the association between usage of medications for the cardiovascular system and falls. (*Unadjusted; No usage of medications for the cardiovascular system was the reference group).

**Figure S55:** Forest plot of the association between usage of walking aid/stick and falls. (*Unadjusted; No walking aid/stick was the reference group).

**Appendix 4 - Summary forest plots for the broad categories of risk factors that could not be included in the meta-analysis.**

**Figure S56:** Summary forest plot of the association between sociodemographic factors and falls.

**Figure S57:** Summary forest plot of the association between environmental factors and falls.

**Figure S58:** Summary forest plot of the association between physical and/or mental health conditions and falls.

**Figure S59:** Summary forest plot of the association between medical interventions and falls.
